# The UIP/IPF fibroblastic focus is a collagen biosynthesis factory embedded in a distinct extracellular matrix

**DOI:** 10.1172/jci.insight.156115

**Published:** 2022-08-22

**Authors:** Jeremy A. Herrera, Lewis Dingle, M. Angeles Montero, Rajamiyer V. Venkateswaran, John F. Blaikley, Craig Lawless, Martin A. Schwartz

**Affiliations:** 1 The Wellcome Centre for Cell-Matrix Research and; 2 Blond McIndoe Laboratories, University of Manchester, Manchester Academic Health Science Centre, Manchester, United Kingdom.; 3 Department of Histopathology, Manchester University National Health Service Foundation Trust, Manchester, United Kingdom.; 4 Faculty of Biology, Medicine and Health, University of Manchester, Manchester Academic Health Science Centre, Manchester, United Kingdom.; 5 Department of Transplant, Manchester University National Health Service Foundation Trust, Manchester, United Kingdom.; 6 Yale Cardiovascular Research Center and; 7 Departments of Internal Medicine (Cardiology) and Cell Biology, Yale School of Medicine, New Haven, Connecticut, USA.; 8 Department of Biomedical Engineering, Yale School of Engineering & Applied Science, New Haven, Connecticut, USA.

**Keywords:** Pulmonology, Extracellular matrix, Fibrosis, Proteomics

## Abstract

Usual interstitial pneumonia (UIP) is a histological pattern characteristic of idiopathic pulmonary fibrosis (IPF). The UIP pattern is patchy with histologically normal lung adjacent to dense fibrotic tissue. At this interface, fibroblastic foci (FF) are present and are sites where myofibroblasts and extracellular matrix (ECM) accumulate. Utilizing laser capture microdissection-coupled mass spectrometry, we interrogated the FF, adjacent mature scar, and adjacent alveoli in 6 fibrotic (UIP/IPF) specimens plus 6 nonfibrotic alveolar specimens as controls. The data were subjected to qualitative and quantitative analysis and histologically validated. We found that the fibrotic alveoli protein signature is defined by immune deregulation as the strongest category. The fibrotic mature scar classified as end-stage fibrosis whereas the FF contained an overabundance of a distinctive ECM compared with the nonfibrotic control. Furthermore, FF were positive for both *TGFB1* and *TGFB3*, whereas the aberrant basaloid cell lining of FF was predominantly positive for *TGFB2*. In conclusion, spatial proteomics demonstrated distinct protein compositions in the histologically defined regions of UIP/IPF tissue. These data revealed that FF are the main site of collagen biosynthesis and that the adjacent alveoli are abnormal. This essential information will inform future mechanistic studies on fibrosis progression.

## Introduction

Idiopathic pulmonary fibrosis (IPF) is a progressive fibrotic lung disease characterized by excessive deposition of extracellular matrix (ECM). IPF histology is consistent with the usual interstitial pneumonia (UIP) pattern. UIP is a fibrotic lung disease, marked by subpleural and paraseptal fibrosis alternating with patchy areas of morphologically normal lung, fibroblastic foci (FF), and honeycomb pattern elsewhere (1). Although UIP histology is the hallmark of IPF (a prototype lung disease of unknown cause), UIP also manifests in other fibrotic lung conditions of unknown cause, such as sarcoidosis, connective tissue disease, and nonspecific interstitial pneumonia, as well as lung fibrosis due to known causes, such as pneumoconiosis and hypersensitivity pneumonitis (1–3). The presence of UIP, independent of disease origin, is associated with rapid disease progression (4).

The areas between fibrosis and morphologically normal lung typically contain fibroblastic growths of variable size termed the FF. These structures are sites of myofibroblast accumulation within a pathological ECM, consistent with the notion that fibrosis spreads from the FF into uninvolved alveoli (5). A 3-dimensional reconstruction of the FF reveals both complex continuous structures and discrete lesions of variable shape (6, 7). IPF is generally considered a fibro-proliferative disease; however, several reports show that the myofibroblasts within the FF are not proliferative (8–12), suggesting that these myofibroblasts serve other biological functions.

It is proposed that FF form due to repetitive epithelial injury and damage responses leading to myofibroblast accumulation (13). The origin of the myofibroblasts within the FF remains an active area of investigation. Available literature suggests that resident lung fibroblasts alone are insufficient but that damaged and stressed alveolar epithelial cells transitioning into myofibroblasts via epithelial-mesenchymal transition (EMT) also contribute (14). Progenitor cells (resident or circulating) may also differentiate into myofibroblasts to promote these lesions (15–17). A general consensus is that the FF and surrounding tissue are an invasive front where myofibroblasts contract, synthesize collagen, and progress toward adjacent uninvolved alveoli (5, 11). Thus, a deeper understanding of FF is needed to determine their biological functions and role in fibrosis progression.

Recently, our group has developed a laser capture microdissection-coupled mass spectrometry (LCM-MS) technique to characterize the ECM of fixed and stained human lung tissue (18), which we now apply to the FF and surrounding tissue. Here, we present a comprehensive molecular tissue atlas that defines cell surface and ECM proteins and conduct pathway analysis of histologically defined regions of the UIP/IPF lung in an effort to understand its pathogenesis.

## Results

### Spatial resolution of the UIP/IPF fibrotic front identifies distinct protein signatures.

**Figure 1:**
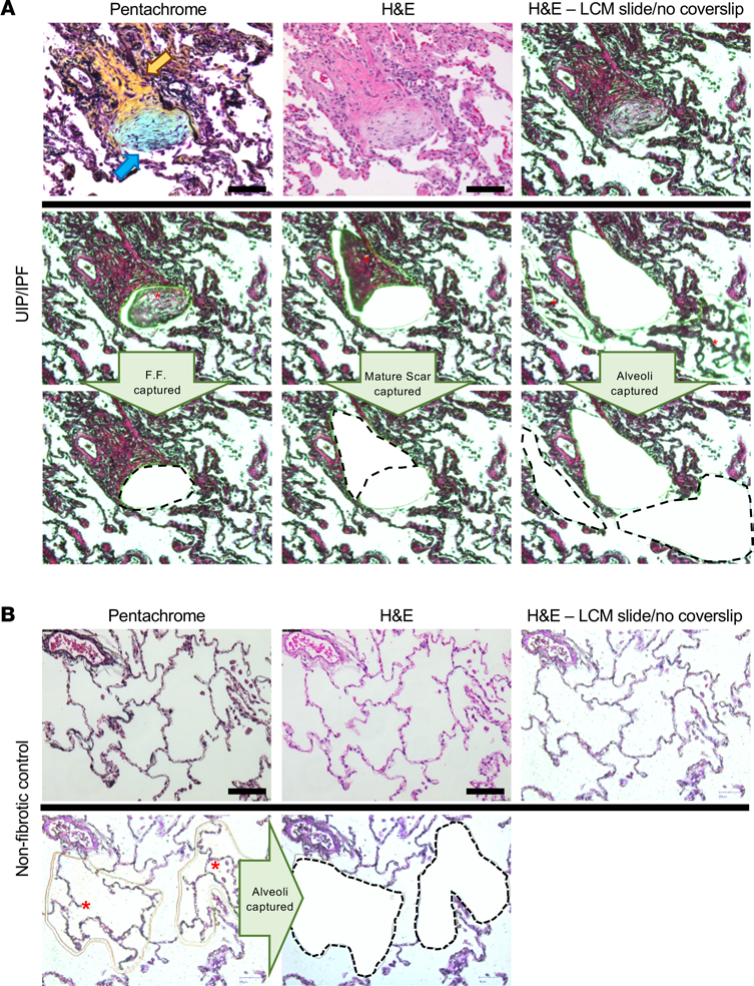
LCM of the FF, mature scar, and adjacent alveoli in a UIP/IPF specimen. (**A**) FFPE specimens were serially sectioned at 5 μm and stained with pentachrome (top left) or H&E (the other 8 panels). Notice that pentachrome stains the FF (hallmark lesion in UIP/IPF) in the color blue (blue arrow), while the mature scar tissue appears yellow in color (yellow arrow). We individually captured the FF (left middle and lower panels), the mature scar tissue (mid-middle and lower panels), and the adjacent alveoli (right middle and lower panels) for MS preparation and analysis. (**B**) Similarly, a nonfibrotic control specimen showing microdissection of alveoli. Scale bar: 100 μm.

Figure 1A shows an example of our LCM approach demonstrating precise cut and capture of the FF, adjacent mature scar, and adjacent alveoli in a fibrotic specimen. Pentachrome stain identifies immature collagen (highlighting the FF) via the blue color while the mature scar tissue appears yellow. We performed LCM on 6 UIP/IPF specimens, capturing 3 regions per specimen: fibrotic alveoli, FF, and mature scar (a total of 18 samples). We captured alveoli from 6 nonfibrotic controls (morphologically normal tissue adjacent to tumors during lung resection) (Figure 1B). For this preparation (a total of 24 samples), we collected tissue volumes of roughly 0.1 mm^3^ per sample, which were then processed for MS. This procedure involved detergent-based heat retrieval, physical disruption, and an in-column trypsin digest system to maximize peptide yield, as we did previously (18).

**Figure 2:**
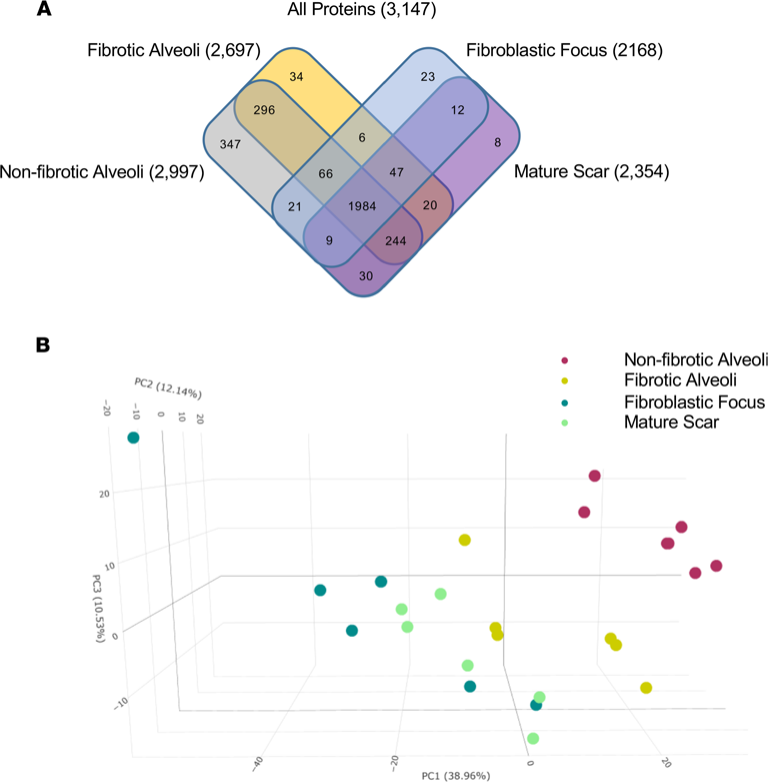
Spatial proteomic analysis of UIP/IPF. UIP/IPF specimens were subjected to LCM-MS to collect mature scar, FF, and fibrotic alveoli (*n* = 6 UIP/IPF specimens). In addition, LCM-MS was performed to collect alveoli from nonfibrotic controls (*n* = 6 nonfibrotic specimens). (**A**) Venn diagram showing all proteins found in each region. (**B**) A 3-dimensional PCA showing that the FF (dark green dots) is most distant from nonfibrotic alveoli (pink dots), with the mature scar (light green dots) returning toward fibrotic alveoli (yellow dots).

In our qualitative analysis approach, we considered a protein present within a sample if it was detected in 3 or more of the 6 samples per group. Using this threshold, qualitative analysis of the MS data showed that we detected 3147 proteins, with the greatest number in the nonfibrotic alveoli control (Figure 2A; a full list of proteins in Supplemental Data 1; supplemental material available online with this article; https://doi.org/10.1172/jci.insight.156115DS1). A 3-dimensional principal component analysis (PCA) based on quantitative MS data (a differential analysis of the proteins using MSqRob v0.7.7; ref. 19) showed that each region uniquely clustered (Figure 2B). First, we saw a clear separation of protein signatures in the nonfibrotic alveoli control (red dots) versus all the fibrotic samples (all other dots), including the structurally intact fibrotic alveoli (yellow dots). Second, we found that the FF cluster (dark green dots) was the furthest from the nonfibrotic alveoli control cluster (red dots). The mature scar is intermediate between the FF and fibrotic alveoli. The protein signatures thus suggest that the UIP/IPF fibrotic front is a distinct environment showing regional changes associated with fibrosis progression.

### The fibrotic alveoli are enriched with immune-regulatory proteins.

**Figure 3:**
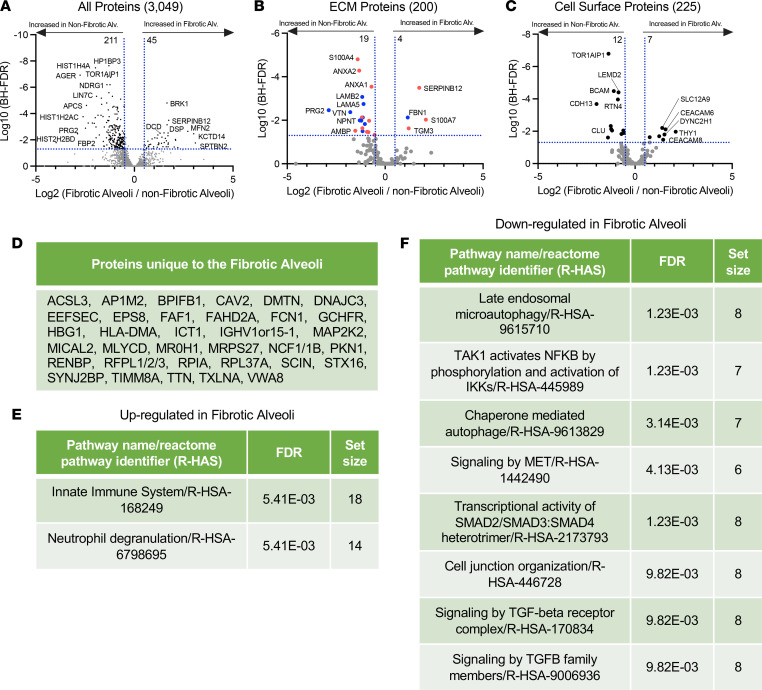
Immune dysregulation defines fibrotic alveoli. (**A**–**C**) Volcano plots comparing fibrotic alveoli and nonfibrotic alveoli control showing the negative natural log of the FDR values plotted against the base 2 log (fold change) for each protein. The data in **A** are for all proteins, whereas data in **B** were matched against the Human Matrisome Project (34) and **C** were matched against the Cell Surface Protein Atlas (39). (**D**) Proteins unique to fibrotic alveoli. Reactome pathways showing the most (**E**) upregulated or (**F**) downregulated for fibrotic alveoli compared with nonfibrotic alveoli control.

Previous reports suggest that the alveoli in IPF are abnormal (20–23); instead, the transition from normal lung to fibrosis shows early pathological features, such as airway remodeling with immune cell infiltration (T cells, B cells, and macrophages). Therefore, we compared fibrotic alveoli with nonfibrotic alveoli controls in more detail. We found 45 proteins that were higher in fibrotic alveoli than 211 proteins that were higher in nonfibrotic alveoli control (Figure 3A). Table 1 lists the top 15 proteins significantly upregulated and downregulated (the full list is in Supplemental Data 2).

Ontologies of the deregulated proteins led us to speculate that abnormal cell migration/invasion within the fibrotic alveoli may contribute to fibrosis progression. The most increased protein, spectrin β non-erythrocyte 2 (*SPTBN2*), is elevated in a variety of cancers and promotes cancer migration via a PI3K/AKT signaling pathway (24). Other increased proteins include *S100A7*, *FTL*, *SERPINB12*, *SHC1*, and *SBSN*, which have characterized roles in cell migration/invasion (25–29). *BRK1* (also known as *HSPC300*) is a regulator of the WAVE complex that controls actin assembly and cell mobility (30). On the other hand, proteins downregulated in the fibrotic alveoli also fit with a role for abnormal cell migration/invasion. For instance, cadherin-13 (*CDH13*) inhibits cancer cell invasion (31) and is decreased in fibrotic alveoli. Arf-GAP with coiled coil (*ACAP1*) regulates membrane trafficking and integrin adhesion complexes (32). Protein lin-7 homolog C (*LIN7C*) is involved in tight junction formation (33). Derangement of these proteins may likely affect cell migration/invasion, which appears to be a mechanistic theme in the fibrotic alveoli.

We further separated our data to identify ECM proteins (Figure 3B) that differ between fibrotic alveoli and nonfibrotic alveoli control by matching our data set to the Human Matrisome Project (http://matrisomeproject.mit.edu) (34). Bone marrow proteoglycan 2 (*PRG2*) has been shown to be suppressed in cancer, has antiinflammatory roles (35), and is lower in fibrotic alveoli. Fibrotic alveoli have higher levels of transglutaminase 3 (*TGM3*). This protein cross-links components of the ECM, which increases matrix rigidity but has also been linked to EMT and AKT signaling pathway in colorectal cancer (36). *PTEN* is a potent negative regulator of PI3-lipids and AKT, which promote both cell invasion and survival. AKT signaling is activated in IPF (37, 38); thus, the concept that changes in the ECM might drive this pathway warrants further study.

Utilizing the Cell Surface Protein Atlas (http://wlab.ethz.ch/cspa/) (39), we matched our data set to the Surfaceome to determine which cell surface receptors are altered in the fibrotic alveoli (Figure 3C). Both the cell-cell adhesion protein *CEACAM6* and the GPI-linked protein *THY1* were increased in the fibrotic alveoli. Interestingly, both are involved in immune cell regulation and are overexpressed in malignancies (40, 41). *THY1* is also implicated in regulating integrin function in the context of fibrosis (42). Interestingly, *TOR1AIP1*, a protein of the nuclear membrane, was decreased in the fibrotic alveoli. Variants of this gene have been identified in limb-girdle muscular dystrophy, and *TOR1AIP1* knockout in striated muscle results in muscle weakness (43, 44). Our qualitative data identified 34 proteins detected only in fibrotic alveoli (Figure 3D), whereas 347 proteins were unique to nonfibrotic alveoli control (Supplemental Data 3). *ACSL3*, which is unique to the fibrotic alveoli, is elevated in pancreatic ductal carcinoma, where it correlates with increased fibrosis (45).

We next used the unbiased Reactome pathway analysis to identify pathways perturbed in fibrotic alveoli. The 2 significantly upregulated categories in fibrotic alveoli were innate immune system and neutrophil degranulation (Figure 3E), consistent with immune involvement in progression of fibrosis (46). Downregulated pathways include late endosomal microautophagy, several categories related to TGFB signaling, and cell junction organization (Figure 3F). Downregulated TGFB signaling at this early, prefibrotic stage would be consistent with the elevated inflammatory status. These data suggest that early events toward disease progression occur well before morphological changes are evident.

### Spatial organization of alveolar epithelial cells and TGFB in UIP/IPF.

**Figure 4:**
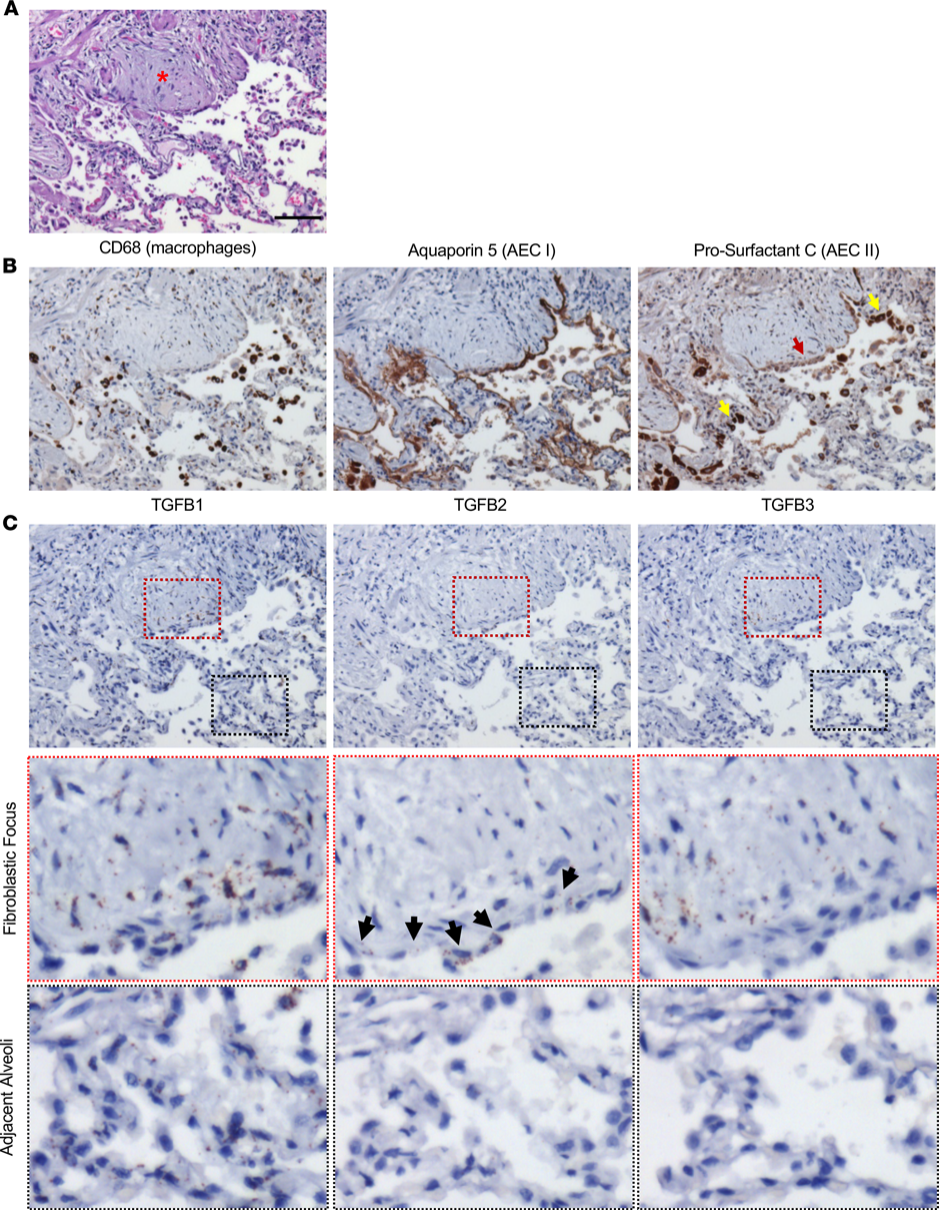
Distribution of type I and II alveolar epithelial cells and TGFB1–3 in the FF and adjacent alveoli. A UIP/IPF specimen was serially sectioned and histologically stained for (**A**) H&E (red asterisk denotes the FF). (**B**) Immunostain for macrophages (*CD68*), AECI (AQ5), and AECII (pSC). Notice that the epithelial lining of the FF has a faint pSC stain (red arrow) yet strong positive staining elsewhere (yellow arrow). (**C**) RNA in situ hybridization for *TGFB1–3*. Notice the epithelial lining of the FF with marked positivity for *TGFB2* (black arrows). Scale bar: 100 μm (**A**–**C**) (*n* = 5 UIP/IPF specimens, representative image shown).

Based on our finding that the UIP/IPF alveolus was abnormal, we next sought to determine the distribution of macrophages, type I alveolar epithelial cells (AECI), and type II alveolar epithelial cells (AECII) within the fibrotic alveoli and FF in UIP/IPF specimens (*n* = 5 UIP/IPF specimens). To distinguish between AECI and AECII, we stained for aquaporin 5 (AQ5) to identify AECI and prosurfactant C (pSC) to identify AECII (47–49). Increased macrophages were previously reported in the IPF lung (21), and we indeed found an accumulation of macrophages (*CD68*-positive cells) within the airspace adjacent to the FF (Figure 4, A and B). Of interest, we found expression of both AQ5 and pSC within the epithelial cells lining the FF, a lining that was intentionally avoided or laser ablated when collecting the FF and adjacent fibrotic alveoli for MS. These cells were previously defined as aberrant basaloid cells (not found in control lung), and we now term this as the aberrant basaloid cell lining (50). Interestingly, the aberrant basaloid cell lining showed a remarkable reduction of pSC stain (red arrow) compared with adjacent cells (yellow arrow). Within the adjacent alveoli, we detected both AECI and AECII along the outer lining of the thickening alveoli. However, we speculate that other cell types (e.g., fibroblasts and progenitor cells) accumulate within the interstitium to cause the thickening.

We next sought to spatially characterize TGFB expression within the fibrotic alveoli and FF based on the changes in TGFB pathways in our reactome pathway analysis. Utilizing RNAscope (in situ RNA hybridization), we identified a potentially unique expression pattern for *TGFB1*, *TGFB2*, and *TGFB3*. Prior single-cell RNA-Seq (scRNA-Seq) data demonstrated that *TGFB1* is broadly expressed in a variety of cells (epithelial, mesenchymal, and immune cells) in IPF specimens. By contrast, *TGFB2* expression is restricted to epithelial cells, and *TGFB3* is highly expressed in mesenchymal cell lineages (51). Our RNAscope data showed that *TGFB1* was also widely expressed in cells within the FF, aberrant basaloid cell lining, macrophages, and adjacent alveoli (Figure 4C). *TGFB2* was predominantly expressed along the aberrant basaloid cell lining but not in adjacent alveoli, suggesting that this lining (positive for both pSC and AQ5) is distinct (Figure 4C, panels with black arrows pointing to the lining). Finally, *TGFB3* was largely within the FF (Figure 4C). Thus, our results are in good agreement with reported scRNA-Seq findings. Additional images of fibrotic specimens (*n* = 5 UIP/IPF specimens total) are in Supplemental Figures 1 and 2. Nonfibrotic controls can be found in Supplemental Figure 3. We note that *TGFB1* was expressed in a variety of cells in nonfibrotic control (like the fibrotic alveoli) and that *TGFB2* and *TGFB3* expressions were less pronounced.

### The mature scar is consistent with end-stage fibrosis.

**Figure 5:**
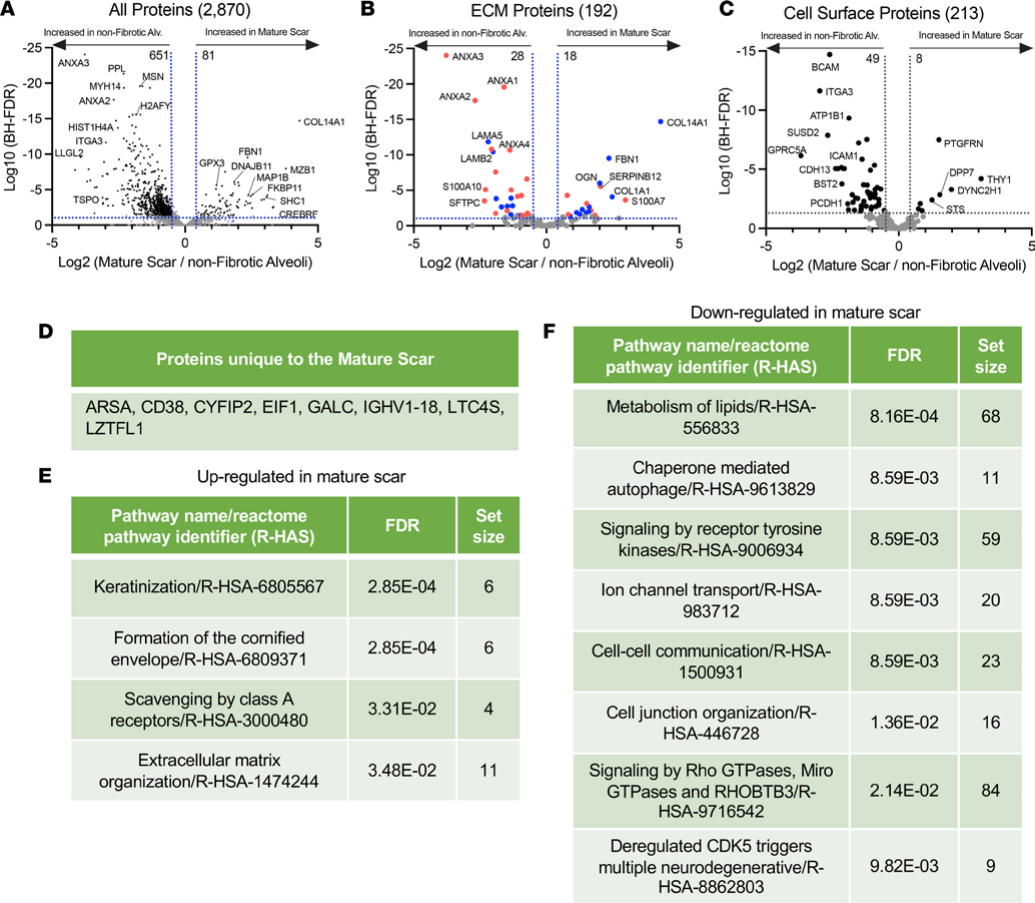
End-stage fibrosis defines mature scar. (**A**–**C**) Volcano plots comparing mature scar to nonfibrotic alveoli control showing the negative natural log of the FDR values plotted against the base 2 log (fold change) for each protein. The data in **A** are for all proteins, whereas data in **B** were matched against the Human Matrisome Project (34) and **C** were matched against the Cell Surface Protein Atlas (39). (**D**) Proteins unique to mature scar. Reactome pathways showing the most (**E**) upregulated or (**F**) downregulated for mature scar compared with nonfibrotic alveoli control.

A comparison of the mature scar with nonfibrotic alveoli control identified 81 proteins increased and 651 proteins decreased in the mature scar compared with nonfibrotic alveoli control (Figure 5A; a full list in Supplemental Data 2). The endoplasmic reticulum chaperone protein *MZB1* was highly elevated in the mature scar, consistent with a report that MZB1-positive plasma B cells (that coexpress *CD38*) are found in end-stage lung and skin fibrosis (52). Supporting possible B cell involvement, *CD38* was among the uniquely expressed proteins in mature scar tissue (Figure 5D). The epithelial polarity and scaffolding protein *LLGL2* was decreased in mature scar, consistent with its high expression in polarized epithelial cells and its loss during EMT (53).

We again matched our data set with the Matrisome project (Figure 5B). Multiple annexin family proteins were decreased in mature scar. Annexin A1 (*ANXA1*) has antiinflammatory effects, and its loss of function exacerbates inflammation in bleomycin-induced lung injury models (54). *ANXA3* is decreased in UIP/IPF but reportedly promotes inflammation (55), whereas *ANXA2* is antiinflammatory (56). Among collagen proteins, *COL14A1* was the most highly upregulated in mature scar. This gene was also a marker for a fibroblast cluster found in bleomycin-induced lung injury by scRNA-Seq (57). Interestingly, collagen XIV is elevated in high–mechanical stress environments, where it regulates fibrillogenesis (58).

We also matched our data set to the Surfaceome (Figure 5C). The transmembrane Ig superfamily protein *PTGFRN* was strongly increased in mature scar. This protein was previously shown to be increased in IPF and in bleomycin-induced lung injury (59). By contrast, the laminin receptor *BCAM* was decreased in mature scar, consistent with decreased expression of laminin proteins in this region (60). Reactome pathway analysis demonstrated that the categories most strongly upregulated in mature scar were keratinization and formation of the cornified envelope (Figure 5E), whereas metabolism of lipids was most strongly decreased (Figure 5F). These changes likely reflect the heavily cross-linked, rigid structure and low metabolic activity of end-stage fibrosis (61).

### The FF shows elevated collagen biosynthesis.

**Figure 6:**
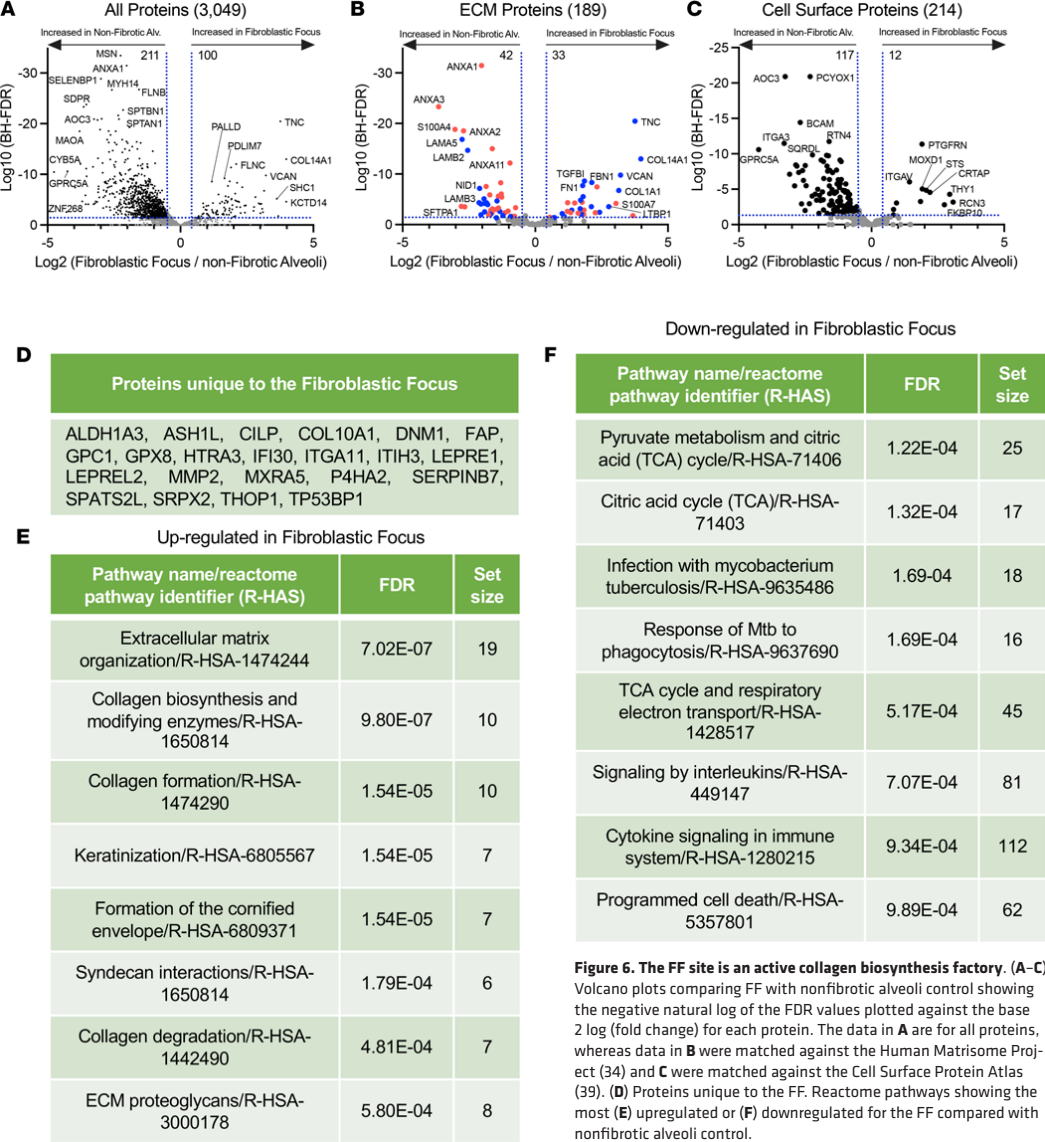
The FF site is an active collagen biosynthesis factory. (**A**–**C**) Volcano plots comparing FF with nonfibrotic alveoli control showing the negative natural log of the FDR values plotted against the base 2 log (fold change) for each protein. The data in **A** are for all proteins, whereas data in **B** were matched against the Human Matrisome Project (34) and **C** were matched against the Cell Surface Protein Atlas (39). (**D**) Proteins unique to the FF. Reactome pathways showing the most (**E**) upregulated or (**F**) downregulated for the FF compared with nonfibrotic alveoli control.

A previous study using LCM of the FF coupled with RNA-Seq and a novel weighted gene coexpression network analysis showed elevation of genes associated with cell cycle, inflammation/differentiation, translation, and cytoskeleton/cell adhesion (62). Our analysis identified 100 proteins increased and 211 decreased in the FF compared with nonfibrotic alveoli control (Figure 6A; a full list in Supplemental Data 2). These data were also matched to both the Matrisome project and the Surfaceome (Figure 6, B and C). The 22 proteins uniquely expressed in the FF are shown in Figure 6D.

Collagen biosynthesis is a complex, multistep process that involves collagen hydroxylation, triple helix formation, cross-linking/maturation, secretion via a specific pathway, and uptake/turnover (reviewed in ref. 63). Collagen is also posttranslationally modified by prolyl-4-hydroxylases (P4HA proteins) and lysyl hydroxylases (PLOD proteins) to promote its stability and function. Consistent with collagen posttranslational modifications, the prolyl-4-hydroxylase *P4HA2* is unique to the FF. The lysyl hydroxylase *PLOD2* is unique to both the FF and mature scar, while *PLOD1* is significantly increased in the FF.

The next step in collagen biosynthesis is the formation of the triple helix. *FKBP10*, which plays a critical role in triple helix formation, is increased in the FF, as is *SERPINH1* (*HSP47*), which functions as an essential collagen-specific chaperone needed for collagen synthesis via effects on triple helix formation and modification (63, 64). In addition, prolyl-3-hydroxylase 3 (*LEPREL2*) is required for proper collagen folding and assembly (65) and is unique to the FF. Collagen is cross-linked/matured by a host of enzymes such as the TGM family. *TGM1* and *TGM3* are increased in the FF. Last, collagen uptake is mediated by the integrin subunits α-1, -2, -10, and -11 and β-1, whereas collagen cleavage is mediated by MMPs. *MMP14* was increased and *MMP2* uniquely expressed in the FF. Integrin α-11 subunit (*ITGA11*), a type collagen I–specific receptor, is unique to the FF, while integrin α-1 (*ITGA1*) and β-1 (*ITGB1*) are decreased. The consequences of these changes are unknown, but effects on collagen fibril organization or turnover are likely.

Reactome pathway analysis showed that FF were indeed enriched for collagen biosynthesis and modifying enzymes, collagen formation, and collagen degradation (Figure 6E), which is in accord with prior data showing that the FF uniquely stains with pro-collagen (active collagen synthesis) (66–68). Interestingly, pyruvate metabolism and citric acid (TCA) cycle were downregulated in FF, which may correlate with low cell proliferation in this region (Figure 6F). These data strongly suggest that the FF is the main site of active collagen biosynthesis.

### The FF experience a dramatic ECM switch.

**Figure 7:**
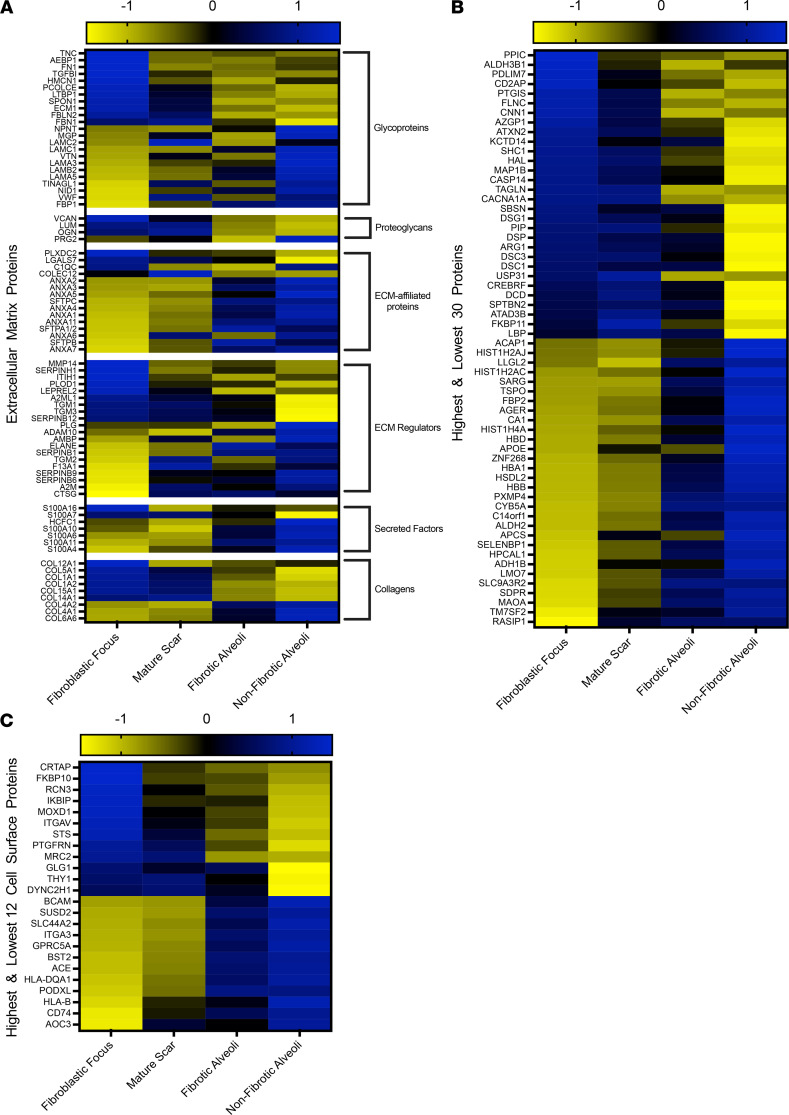
The FF has a unique ECM signature. Shown are heatmaps to demonstrate the expression of (**A**) ECM proteins, (**B**) the highest and lowest 30 proteins (excluding ECM and cell surface proteins), and (**C**) the highest and lowest 12 cell surface proteins in the FF, mature scar, fibrotic alveoli, and nonfibrotic alveoli. Blue indicates a protein increase and yellow indicates a protein decrease.

We next aligned all of our data (FF, mature scar, fibrotic alveoli, and nonfibrotic alveoli control) to derive a heatmap of all statistically significant ECM proteins matched to the 6 matrisome categories (glycoproteins, proteoglycans, ECM-affiliated proteins, ECM regulators, secreted factors, and collagens) (Figure 7A). In every case, the ECM proteins comprising the FF showed the greatest difference from nonfibrotic alveoli control. For instance, it is well known that collagen IV, a major constituent of epithelial basement membranes, decreases in IPF (69). We also found that collagen IV was low in both the FF and mature scar and high in both fibrotic alveoli and nonfibrotic alveoli control. Similarly, collagen I was high in both mature scar and FF and low in both fibrotic alveoli and nonfibrotic alveoli control (Figure 7A). Overall, fibrotic alveoli tended to resemble the nonfibrotic alveoli controls, whereas mature scar tended to resemble the FF, with substantial deviations. Examination of the qualitative data to determine the numbers of ECM proteins by categories and per region (Supplemental Figure 4) also revealed no dramatic difference in the types of ECM proteins found within each region. Taken together, these results reveal a dramatic switch of ECM in the FF.

Similarly, we created a heatmap of the highest and lowest 30 statistically most changed proteins (Figure 7B; a full list in Supplemental Data 4). The top 3 proteins expressed in the FF were *PPIC*, *ALDH3B1*, and *PDLIM7*. *PPIC* is associated with protein folding and is upregulated in CCL4-induced liver injury. *PPIC* loss of function was shown to improve liver injury, suggesting it may be involved in fibrosis progression (70). Similarly, *ALDH3B1*, which is involved in oxidative stress, is highly expressed in lung cancer (71). *PDLIM7* regulates cellular senescence (72), a process that likely contributes to IPF (73). Analysis of the 12 highest and lowest statistically changed cell surface proteins (Figure 7C) identified *CRTAP* and *RCN3* as the most increased in the FF. Both genes are critical for collagen biosynthesis (74, 75). According to reports showing that FF are hypoxic (76), FF showed increased steroid sulfatase (*STS*), a gene involved in steroid hormone synthesis that was reported to induce hypoxia inducible factor 1 (*HIF1*) expression (77). We also noted that both integrin-v (*ITGAV*; cell surface protein) and latent TGF-β–binding protein 1 (*LTBP1*; ECM protein) were increased in the FF, consistent with their roles in activating latent TGFB (78). This study provides us a list of some established and many potentially new region-specific proteins to begin exploring their collective functions in the pathogenesis of UIP/IPF.

Last, we sought to compare the FF with mature scar tissue (Supplemental Figure 5; a full list in Supplemental Data 5). The FF showed greater upregulation of proteins involved in collagen biosynthesis and ECM organization. Major ECM proteins highlighted here include *TNC*, *CTHRC1*, *SERPINH1*, *FN1*, *VCAN*, and *COL12A1*, all of which are involved in assembly or stabilization of the fibrotic ECM.

**Figure 8:**
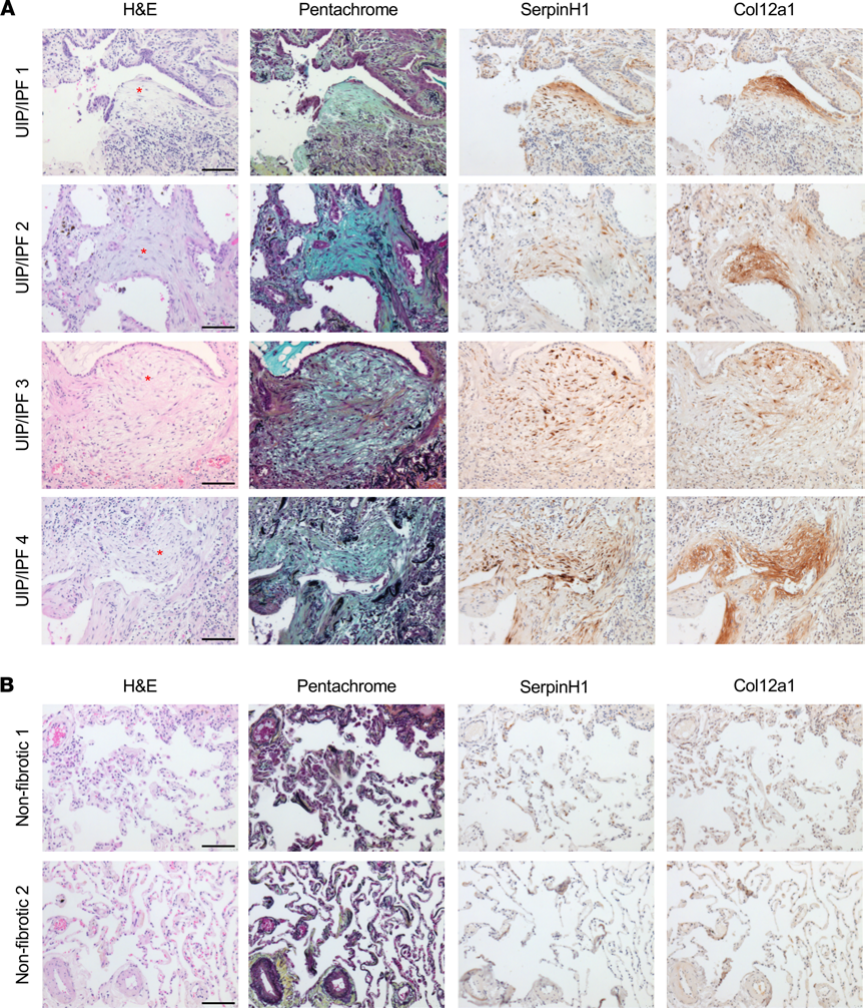
*SERPINH1* and *COL12A1* are enriched in the FF. A total of (**A**) 4 UIP/IPF specimens and (**B**) 2 nonfibrotic control specimens were serially sectioned and stained for H&E, pentachrome, anti-*SERPINH1*, and anti-*COL12A1*. We show a representative FF per specimen (depicted with a red asterisk). Scale bar: 100 μm.

To validate some of these results, we immunostained for *SERPINH1* and *COL12A1* in 4 UIP/IPF specimens and 2 nonfibrotic controls. Consistent with the proteomic analysis, we found both specifically enriched within the FF (Figure 8A and Supplemental Figure 6). Expression of *SERPINH1* (essential for collagen synthesis) within the FF was consistent with its role in collagen biosynthesis. By contrast, neither *SERPINH1* nor *COL12A1* was detected in nonfibrotic controls (Figure 8B).

## Discussion

**Figure 9:**
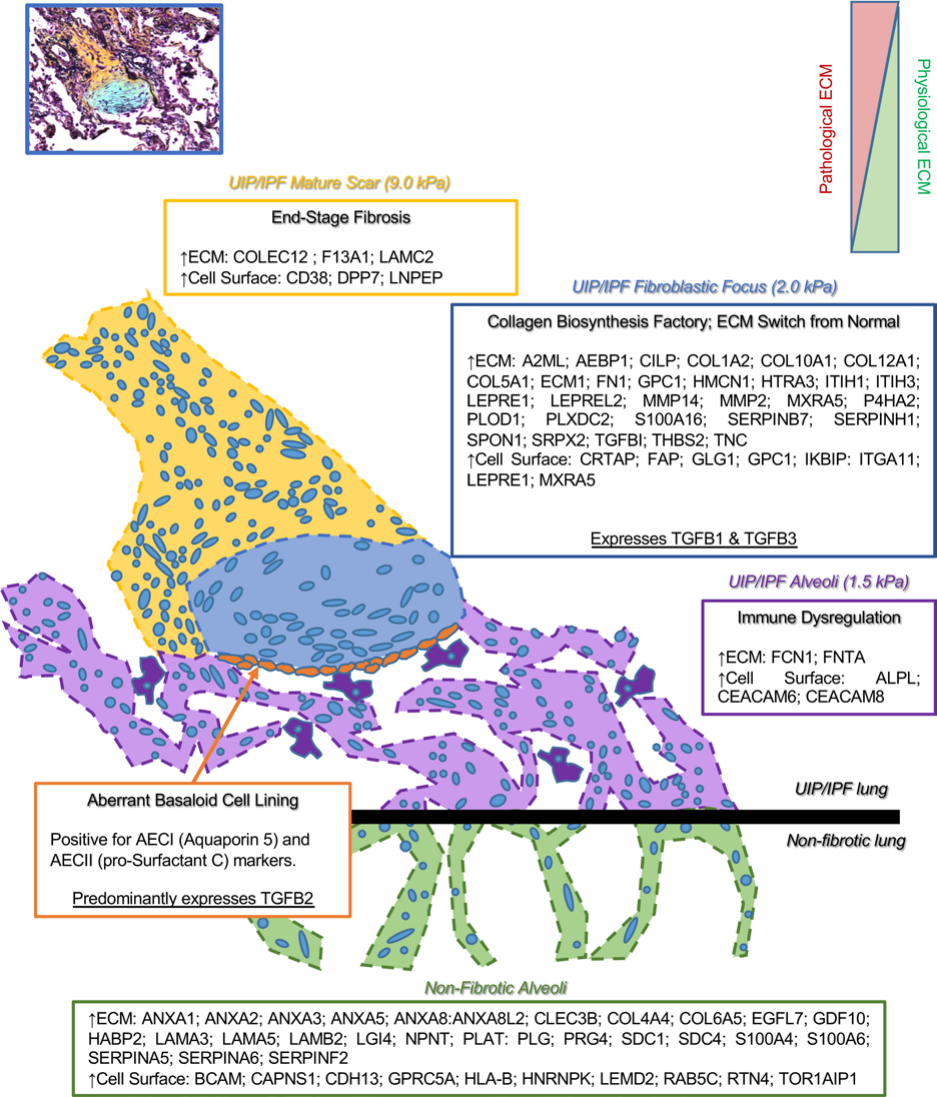
The FF. The lesion of UIP/IPF is termed the FF and can be envisioned as the invasive front. The FF progresses toward adjacent alveoli, leaving behind a dense mature scar tissue. Herein, we provide a list of proteins (ECM and cell surface) that are abundantly and significantly overexpressed per region. We define the FF as a collagen biosynthesis factory that expresses both *TGFB1* and *TGFB3*, while embedded in a unique ECM, a switch from normal. Adjacent to the FF, the mature scar is characterized as end-stage fibrosis, which is stiffer than the rest of the fibrotic tissue. The alveoli adjacent to the FF accumulate macrophages and are defined by immune dysregulation. The epithelial lining along the FF (termed the aberrant basaloid cell lining; ref. 50) is positive for both AECI and AECII markers (AQ5 and pSC, respectively) and predominantly expresses *TGFB2*.

The availability of spatial omics makes it possible to reveal previously inaccessible clinical features of human disease. Herein, we produced spatial proteomic profiles of the UIP/IPF fibrotic front to characterize the protein composition of nonfibrotic alveoli, fibrotic alveoli, the FF, and mature scar. We create a tissue atlas of the UIP/IPF fibrotic front (Figure 9) defining the ECM and cell surface proteins of each region in an effort to inform novel hypotheses about cell/ECM systems in fibrosis progression. Recent work utilizing atomic force microscopy to define mechanical properties of the different zones of UIP/IPF tissue showed that FF (2.0 kPa) were surprisingly as soft as adjacent fibrotic alveoli (1.5 kPa), whereas the mature scar was much stiffer (9.0 kPa) (79). Unfortunately, nonfibrotic controls were not included in that report. The low stiffness of FF was surprising, especially in light of our finding of increased levels of a variety of collagen and ECM proteins. We speculate that the enrichment of glycoproteins and proteoglycans, which have the capacity to hydrate tissues (80), may be responsible for the softness of the FF.

Extensive evidence suggests that normal-appearing alveoli within the UIP/IPF lung are in fact significantly abnormal. RNA-Seq of tissue from different regions of the IPF lung showed that structurally normal IPF tissue had over 1000 differentially expressed genes compared with nonfibrotic tissue (20). Increased immune cell infiltration (innate and adaptive) has also been observed in structurally normal regions of IPF lung (21). A 3-dimensional image analysis of the IPF lung (specifically looking at regions with no evidence of microscopic fibrosis) showed reduced numbers of small airways and thickening of terminal bronchiolar walls, suggesting that early remodeling is crucial to IPF pathogenesis (23). An important unanswered question is whether the changes of the alveoli observed here indicate early steps in fibrosis, which eventually lead to the development of the FF and then mature scar tissue. Alternatively, it may be that the FF and mature scar affect surrounding lung tissue, potentially through secreted factors that act directly, by promoting immune cell infiltration, or by altering lung mechanics. Future studies analyzing spatial proteomics in multiple regions of the uninvolved fibrotic lung are needed to address this question.

The mature scar region in UIP/IPF is consistent with end-stage fibrosis. McDonough et al. (20) similarly sampled regions of the IPF lung from structurally normal to increasing fibrosis (IPF level 1 through 3). They concluded that at the transcriptional level, the transition from level 2 to level 3 (the most fibrotic tissue) involves genes characteristic of end-stage disease (20), consistent with the higher stiffness in these regions (79). In regard to this study, we find some agreements in the proteome as compared with the transcriptome. For instance, *THY1* is decreased at both the RNA (IPF levels 1–3) and protein (fibrotic alveoli, FF, and mature scar) level in fibrotic specimens. *AGER* (the gene for the inflammatory receptor RAGE) is a central mediator of inflammation and is transcriptionally decreased in IPF regions 2 and 3 (not level 1, the most normal), whereas *AGER* protein was suppressed in all fibrotic regions looked at here (20, 81). Similarly, *COL1A1*, *COL1A2*, *COL5A1*, and *COL15A1* were shown to be transcriptionally increased in IPF levels 1–3; however, we only find that these collagen proteins are increased in the FF and mature scar (not the fibrotic alveoli). Although there are substantial deviations, these may be attributed to effects on message translation, protein stability, and ECM assembly that can account for differences with RNA levels, as well as perhaps the low resolution of spatial transcriptomics.

FF are the signature lesions of UIP/IPF (1) and are consistently described as the site of collagen synthesis. Our data defining the protein constituents of this domain lead to 2 major conclusions. First, a large fraction of the proteins necessary for collagen biosynthesis are either increased or uniquely expressed within the FF. The FF region is, therefore, the primary region of active collagen biosynthesis. Second, we provide evidence that the ECM of the FF is completely switched from nonfibrotic alveoli controls, with near-complete replacement of the epithelial basement membrane with an interstitial collagenous ECM. We speculate that the synthesis and assembly of matrix within the FF may be critical for migration of myofibroblasts or their precursor into uninvolved airspaces. Local inhibition of collagen biosynthesis machinery may, therefore, be a pharmaceutical approach to stopping fibrosis progression.

The aberrant basaloid cells lining the FF are a unique feature, as these cells are not found in controls. In fact, they only comprise 3.5% of the IPF epithelial population by scRNA-Seq (50). Histologically, the cells within this lining are flat or cuboidal with centrally located and bland-looking nuclei. Our group and others have reported that the expression of procollagen I (active collagen synthesis) is strongest in fibroblasts adjacent to the airway (66, 82), which is immediately adjacent to the aberrant basaloid cell lining. Thus, it is likely that *TGFB2* acts locally on these fibroblasts to promote collagen biosynthesis. Similarly, the expression of *TGFB1* and *TGFB3* within the FF could be acting to induce the aberrant basaloid cell lining. Although TGFB isoforms bind similar receptors and signal through the same pathways (83), the spatial organization of TGFB along the fibrotic front warrants further investigation.

Although UIP is the characteristic histological pattern in IPF, the UIP pattern has been reported in other lung diseases such as sarcoidosis, connective tissue disease, nonspecific interstitial pneumonia, pneumoconiosis, and hypersensitivity pneumonitis. In our 6 specimens, all had a UIP pattern (5 with an IPF diagnosis and 1 with a sarcoidosis diagnosis). We found that the sarcoidosis specimen was not an outlier in our data set, and its removal did not substantially alter the results (Supplemental Figure 7). Although the small sample size is a limitation to our study, this outcome raises the possibility that the UIP pattern may be similar, independent of the origin.

Recently, a gene expression analysis (utilizing Nanostring Technologies fibrosis-specific gene panel) was performed on the FF in UIP/IPF and sarcoidosis as compared with nonfibrotic lung (84). We find high agreement with this study. They reported increased *THBS2* and *FAP*, which were both unique to the FF at the protein expression level. They showed increased *COL1A1*, *COL1A2*, *COL5A1*, *COL6A3*, and *COL14A1* mRNA, all of which were increased in the FF at the protein expression level. Decreased in the transcriptome were *LPCAT1*, *FASN*, *CYBB*, *PGK1*, *PECAM1*, *CD36*, and *CAT*, all of which were decreased by protein expression level. Of interest, mRNA for *GOT2*, *NDUFS3*, *PGM1*, *UQCRB*, *ALDH7A1*, *HADH*, and *PSMD13* were significantly increased, but these were decreased at the protein expression level. These results likely reflect effects on translational efficiency of protein turnover (85, 86). The authors concluded that FF are remarkably similar, independent of origin (UIP/IPF or sarcoidosis), consistent with this being a site of collagen biosynthesis.

In another report, LCM-coupled RNA-Seq was performed on the FF in UIP/IPF (62). This work used a novel weighted analysis to predict pathways of interest to the FF. However, these data do not agree well with our findings. For instance, the strongest featured node within their network analysis includes *RHOA/RAC1/ROCK* signaling and their regulators *ARHGEF1/ARHGEF2/AKAP13*. By contrast, these proteins were significantly downregulated in the FF compared with nonfibrotic alveoli controls. Their next positively correlated node is *JAK1/STAT1/STAT3*; again, both *STAT1/STAT3* proteins were decreased in the FF, while *JAK1* was undetected. These studies used very different methods and comparisons; thus, firm conclusions await a more direct experimental comparison. If these findings are confirmed, changes in protein translation, stability, or detection could account for these differences.

Herein we create an unbiased tissue atlas of the UIP/IPF fibrotic front utilizing LCM-MS. We demonstrate that there are regional changes in protein signatures associated with fibrosis progression and provide detailed lists of proteins that suggest hypotheses concerning UIP/IPF pathology and progression. The finding that uninvolved alveoli within the fibrotic lung are highly abnormal suggests that this region may contain novel targets for therapeutic intervention.

## Methods

### Histological staining.

Human lung specimens were FFPE and sectioned at 5 μm on appropriate slides. H&E staining was achieved using an automated stainer (Leica XL) at the University of Manchester’s Histology Core. Briefly, the slides were heated at 60°C for 20 minutes, dewaxed with xylene, dehydrated by alcohol treatment, and rinsed in tap water. Slides were then hematoxylin stained for 2 minutes, acid-alcohol treated, and stained for 1 minute in eosin. Slides were then washed in 100% ethanol and allowed to air-dry. These slides were stored at 4°C for up to 1 week while performing LCM. For pentachrome staining, we used a modified Russel-Movats pentachrome stain protocol, which we have previously described in detail (18).

For IHC, we utilized the Novolink Polymer Detection Systems (Leica, catalog RE7200-CE) as previously described in detail (66). Deparaffinized and rehydrated 5 μm FFPE sections were subjected to antigen heat retrieval using citrate buffer (Abcam, catalog ab208572) in a preheated steam bath (100°C) for 20 minutes, before cooling to room temperature for 20 minutes. Slides were then treated with 3%–4% hydrogen peroxide (Leica Biosystems, catalog RE7101) for 10 minutes, blocked in SuperBlock buffer (TBS; Thermo Scientific, catalog 37581) for a minimum of 1 hour, and probed with primary antibodies overnight at 4°C in 10% SuperBlock solution in Tris-buffered saline Tween 20 solution (TBS-T; pH 7.6). Primary antibodies used were as follows: *SERPINH1* (titer 1:20,000; Abcam, catalog ab109117) and *COL12A1* (titer 1:200; Abcam, catalog ab21304).

The following day, the specimens were subjected to Novolink Polymer Detection Systems (Leica Biosystems, catalog RE7270-RE) per the manufacturer’s recommendations, with multiple TBS-T washes. Sections were developed for 5 minutes with DAB Chromagen (Cell Signaling Technology, catalog 11724/5) before being counterstained with hematoxylin, dehydrated through sequential ethanol and xylene, and coverslipped with Permount mounting medium (Thermo Fisher Scientific, catalog SP15).

### RNAscope.

FFPE 5 μm tissue sections were created a day before probe was applied following the manufacturer’s guidelines (Bio-Techne). We utilized RNAscope 2.5 High Definition Brown Assay (catalog 322300) and probed for human *TGFB1* (catalog 489221), *TGFB2* (catalog 489241), and *TGFB3* (catalog 489231) (Bio-Techne). Sections were counterstained with hematoxylin and coverslipped with permount.

### Histological imaging.

Stained slides were imaged using a DMC2900 Leica instrument with Leica Application Suite X software.

### LCM.

To precisely perform this experiment, 3 serial sections were created per specimen. The first section was placed onto a standard glass slide for pentachrome stain, and the next 2 serial sections were placed onto 2 separate Molecular Machines & Industries (MMI) membrane slides (catalog 50102) for H&E staining (see above for staining details). The MMI CellCut Laser Microdissection System can load up to 3 slides. In the first slot, we loaded the first pentachrome-stained slide followed by the 2 H&E-stained MMI slides. We used MMI CellCut software to perform a complete CellScan of the pentachrome slide and H&E slides to allow for careful tissue registration. Using the pentachrome slide as a guide, we then used a closed-shape drawing tool to outline regions of interest. Automated cutting was achieved by adjusting the settings to a laser focus of 350 μm, a 60% laser power, and 50 μm/s speed. Using adhesive MMI transparent caps (catalog 50204) and MMI CapLift technology, we gently lifted dissected specimens onto the adhesive caps, which were stored in –20°C for several weeks until all samples were collected and processed for MS. Ultimately, we used up to 30 sections (in sets of 3) to capture desired volumes (~0.1 mm^3^) per region of the IPF specimen.

### MS sample preparation.

Samples were prepared using a multistep method as previously described in our detailed technical report with minor modifications (18). First, after samples are trypsin digested, instead of eluting in 50% acetonitrile with 0.2% formic acid, we now elute in 30% acetonitrile with 0.2% formic acid. In addition, after those samples are dried by speedvac, we now resuspend in 3% acetonitrile with 0.1% formic acid (instead of 5% acetonitrile with 0.1% formic acid). Last, our final elution after desalting our samples now uses 30% acetonitrile (instead of 50% acetonitrile). These were important as we began experiencing downstream contamination that we speculated was due to high acetonitrile concentrations, a problem that was resolved by lowering acetonitrile.

### Liquid chromatography-coupled tandem MS.

As previously described, peptides were resuspended in 10 μL of 3% acetonitrile, with 0.1% formic acid, and 1 μL was used for evaluation by liquid chromatography-coupled tandem MS using an UltiMate 3000 Rapid Separation LC system (Dionex Corporation) coupled to a Q Exactive HF mass spectrometer (Thermo Fisher Scientific).

### Data availability.

The MS proteomics data have been deposited to the ProteomeXchange consortium via the PRIDE (87) partner repository with the data set identifier PXD029341.

### Statistics.

Raw data were processed using MaxQuant software (v1.6.17.0) (88) against the human proteome obtained from Uniprot (May 2021) (89). Variable modifications were set as methionine oxidation and N-terminal acetylation, with a fixed modification of carbamidomethylation of cysteine. The protein and PSM FDRs were set at 0.01 and “match between runs” was enabled.

Comparative statistical analysis was performed using MSqRob (v0.7.7) (19) in the R environment (v4.1.0) (90). Proteins significantly changing between conditional comparisons were taken at 5% FDR. Reactome pathway analysis of differentially expressed proteins was performed using the R package ReactomePA (1.36.0) (91).

### Study approval.

Tissue from patients who provided written informed consent was collected following University of Manchester Health Research Authority–approved protocols (REC 14/NW/0260; provided by JFB and RVV) for fibrotic specimens during lung transplantation and (REC 20/NW/0302; provided by MAM) for nonfibrotic control specimens. The specimens were diagnosed as IPF having characteristic UIP, FF, and honeycomb pattern as defined by current guidelines (1). Nonfibrotic controls were collected from morphologically normal lung tissue distal to tumors during resection. In this study, we used 5 IPF specimens plus 1 end-stage lung fibrosis specimen from a patient with a history of sarcoidosis with UIP pathology who was clinically treated as patient with IPF before transplantation (Supplemental Table 1). Reexamination of the specimen from this patient collected for our research showed no evidence of sarcoidosis. Instead UIP, FF, and honeycombing pattern was observed. We now collectively term our fibrotic specimens UIP/IPF. This UIP/sarcoidosis specimen was not an outlier, did not alter our results when removed, and was thus kept throughout the analysis (Supplemental Figure 7).

## Author contributions

JAH and MAS conceived and supervised the project. JAH designed and conducted all LCM-MS experiments and CL performed the associated analyses. LD performed the IHC. MAM assisted in characterizing the histological stains and identification of clinical morphologies associated with UIP/IPF. RVV and JFB contributed to reagents. JAH and MAS wrote the manuscript with all authors’ input.

## Supplementary Material

Supplemental data

Supplemental data set 1

Supplemental data set 2

Supplemental data set 3

Supplemental data set 4

Supplemental data set 5

## Figures and Tables

**Table 1:**
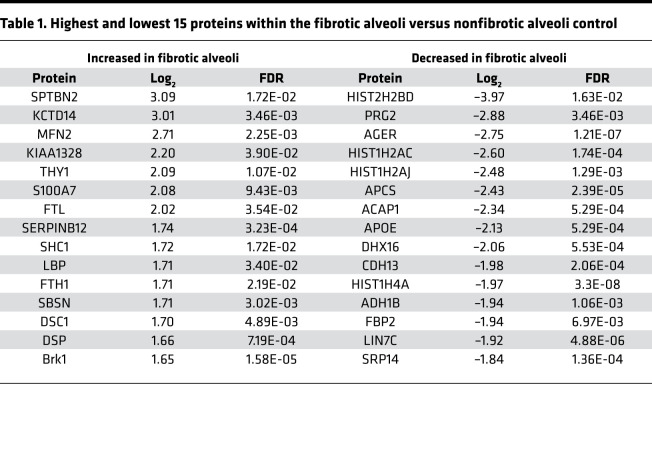
Highest and lowest 15 proteins within the fibrotic alveoli versus nonfibrotic alveoli control

## Version 1 07/19/2022

In-Press Preview

## Version 2 08/22/2022

Electronic publication

## References

[1] Raghu G (2018). Diagnosis of idiopathic pulmonary fibrosis. An official ATS/ERS/JRS/ALAT clinical practice guideline. Am J Respir Crit Care Med.

[2] Collins BF (2018). Sarcoidosis and IPF in the same patient-a coincidence, an association or a phenotype?. Respir Med.

[3] Lynch DA (2018). Diagnostic criteria for idiopathic pulmonary fibrosis: a Fleischner Society White Paper. Lancet Respir Med.

[4] Brown KK (2020). The natural history of progressive fibrosing interstitial lung diseases. Eur Respir J.

[5] Herrera J (2018). Extracellular matrix as a driver of progressive fibrosis. J Clin Invest.

[6] Cool CD (2006). Fibroblast foci are not discrete sites of lung injury or repair: the fibroblast reticulum. Am J Respir Crit Care Med.

[7] Jones MG (2016). Three-dimensional characterization of fibroblast foci in idiopathic pulmonary fibrosis. JCI Insight.

[8] Lomas NJ (2012). Idiopathic pulmonary fibrosis: immunohistochemical analysis provides fresh insights into lung tissue remodelling with implications for novel prognostic markers. Int J Clin Exp Pathol.

[9] Cha SI (2010). Compartmentalized expression of c-FLIP in lung tissues of patients with idiopathic pulmonary fibrosis. Am J Respir Cell Mol Biol.

[10] Vuorinen K (2008). Peroxiredoxin II expression and its association with oxidative stress and cell proliferation in human idiopathic pulmonary fibrosis. J Histochem Cytochem.

[11] Xia H (2017). Calcium-binding protein S100A4 confers mesenchymal progenitor cell fibrogenicity in idiopathic pulmonary fibrosis. J Clin Invest.

[12] Hecker L (2014). Reversal of persistent fibrosis in aging by targeting Nox4-Nrf2 redox imbalance. Sci Transl Med.

[13] King TE (2011). Idiopathic pulmonary fibrosis. Lancet.

[14] Hill C (2019). Epithelial-mesenchymal transition contributes to pulmonary fibrosis via aberrant epithelial/fibroblastic cross-talk. J Lung Health Dis.

[15] Beisang DJ (2020). Single-cell RNA sequencing reveals that lung mesenchymal progenitor cells in IPF exhibit pathological features early in their differentiation trajectory. Sci Rep.

[16] Xia H (2014). Identification of a cell-of-origin for fibroblasts comprising the fibrotic reticulum in idiopathic pulmonary fibrosis. Am J Pathol.

[17] Strieter RM (2009). The role of circulating mesenchymal progenitor cells (fibrocytes) in the pathogenesis of pulmonary fibrosis. J Leukoc Biol.

[18] Herrera JA (2020). Laser capture microdissection coupled mass spectrometry (LCM-MS) for spatially resolved analysis of formalin-fixed and stained human lung tissues. Clin Proteomics.

[19] Goeminne LJE (2018). Experimental design and data-analysis in label-free quantitative LC/MS proteomics: a tutorial with MSqRob. J Proteomics.

[20] McDonough JE (2019). Transcriptional regulatory model of fibrosis progression in the human lung. JCI Insight.

[21] Xu F (2021). The transition from normal lung anatomy to minimal and established fibrosis in idiopathic pulmonary fibrosis (IPF). EBioMedicine.

[22] Figueira de Mello GC (2010). Small airway remodeling in idiopathic interstitial pneumonias: a pathological study. Respiration.

[23] Ikezoe K (2021). Small airway reduction and fibrosis is an early pathologic feature of idiopathic pulmonary fibrosis. Am J Respir Crit Care Med.

[24] Wang P (2021). SPTBN2 regulated by miR-424-5p promotes endometrial cancer progression via CLDN4/PI3K/AKT axis. Cell Death Discov.

[25] Padilla L (2017). S100A7: from mechanism to cancer therapy. Oncogene.

[26] Li Z (2020). Ferritin light chain (FTL) competes with long noncoding RNA Linc00467 for miR-133b binding site to regulate chemoresistance and metastasis of colorectal cancer. Carcinogenesis.

[27] Basson MD (2018). Schlafen 12 interaction with SerpinB12 and deubiquitylases drives human enterocyte differentiation. Cell Physiol Biochem.

[28] Li X (2015). Negative feedback loop between p66Shc and ZEB1 regulates fibrotic EMT response in lung cancer cells. Cell Death Dis.

[29] Pribyl M (2021). Suprabasin-a review. Genes (Basel).

[30] Cai X (2009). Metastatic potential of lung squamous cell carcinoma associated with HSPC300 through its interaction with WAVE2. Lung Cancer.

[31] Andreeva AV (2010). Cadherin 13 in cancer. Genes Chromosomes Cancer.

[32] Vitali T (2019). Arf GAPs: a family of proteins with disparate functions that converge on a common structure, the integrin adhesion complex. Small GTPases.

[33] Straight SW (2006). Mammalian lin-7 stabilizes polarity protein complexes. J Biol Chem.

[34] Naba A (2012). The matrisome: in silico definition and in vivo characterization by proteomics of normal and tumor extracellular matrices. Mol Cell Proteomics.

[35] Carpino G (2019). Matrisome analysis of intrahepatic cholangiocarcinoma unveils a peculiar cancer-associated extracellular matrix structure. Clin Proteomics.

[36] Feng Y (2020). TGM3 functions as a tumor suppressor by repressing epithelial‑to‑mesenchymal transition and the PI3K/AKT signaling pathway in colorectal cancer. Oncol Rep.

[37] Qiu T (2019). PTEN loss regulates alveolar epithelial cell senescence in pulmonary fibrosis depending on Akt activation. Aging (Albany NY).

[38] Miyoshi K (2013). Epithelial Pten controls acute lung injury and fibrosis by regulating alveolar epithelial cell integrity. Am J Respir Crit Care Med.

[39] Bausch-Fluck D (2015). A mass spectrometric-derived cell surface protein atlas. PLoS One.

[40] Pandey R (2019). Carcinoembryonic antigen cell adhesion molecule 6 (CEACAM6) in pancreatic ductal adenocarcinoma (PDA): An integrative analysis of a novel therapeutic target. Sci Rep.

[41] Felley-Bosco E (2019). Editorial: Thy1/CD90 surface glycoprotein: sensor of microenvironment?. Front Cell Dev Biol.

[42] Hu P (2019). Thy-1 in integrin mediated mechanotransduction. Front Cell Dev Biol.

[43] Cossins J (2020). Congenital myasthenic syndrome due to a TOR1AIP1 mutation: a new disease pathway for impaired synaptic transmission. Brain Commun.

[44] Ghaoui R (2016). TOR1AIP1 as a cause of cardiac failure and recessive limb-girdle muscular dystrophy. Neuromuscul Disord.

[45] Rossi Sebastiano M (2020). ACSL3-PAI-1 signaling axis mediates tumor-stroma cross-talk promoting pancreatic cancer progression. Sci Adv.

[46] Desai O (2018). The role of immune and inflammatory cells in idiopathic pulmonary fibrosis. Front Med (Lausanne).

[47] Li A (2013). Deletion of mesenchymal glucocorticoid receptor attenuates embryonic lung development and abdominal wall closure. PLoS One.

[48] Liu X (2020). Effects of different ligands in the Notch signaling pathway on the proliferation and transdifferentiation of primary type II alveolar epithelial cells. Front Pediatr.

[49] Wu J (2017). Characterization of air-liquid interface culture of A549 alveolar epithelial cells. Braz J Med Biol Res.

[50] Adams TS (2020). Single-cell RNA-seq reveals ectopic and aberrant lung-resident cell populations in idiopathic pulmonary fibrosis. Sci Adv.

[51] Sun T (2021). TGFβ2 and TGFβ3 isoforms drive fibrotic disease pathogenesis. Sci Transl Med.

[52] Schiller HB (2017). Deep proteome profiling reveals common prevalence of MZB1-positive plasma B cells in human lung and skin fibrosis. Am J Respir Crit Care Med.

[53] Liu Y (2015). Loss of BRMS1 promotes a mesenchymal phenotype through NF-κB-dependent regulation of Twist1. Mol Cell Biol.

[54] Damazo AS (2011). Endogenous annexin A1 counter-regulates bleomycin-induced lung fibrosis. BMC Immunol.

[55] Cui Y (2021). Identification of key candidate genes involved in the progression of idiopathic pulmonary fibrosis. Molecules.

[56] Dallacasagrande V, Hajjar HA (2020). Annexin A2 in inflammation and host defense. Cells.

[57] Xie T (2018). Single-cell deconvolution of fibroblast heterogeneity in mouse pulmonary fibrosis. Cell Rep.

[58] Berthod F (1997). Differential expression of collagens XII and XIV in human skin and in reconstructed skin. J Invest Dermatol.

[59] Yi H (2021). Knockdown of long non‑coding RNA DLEU2 suppresses idiopathic pulmonary fibrosis by regulating the microRNA‑369‑3p/TRIM2 axis. Int J Mol Med.

[60] Latini FR (2013). DARC (Duffy) and BCAM (Lutheran) reduced expression in thyroid cancer. Blood Cells Mol Dis.

[61] Candi E (2005). The cornified envelope: a model of cell death in the skin. Nat Rev Mol Cell Biol.

[62] Guillotin D (2021). Transcriptome analysis of IPF fibroblastic foci identifies key pathways involved in fibrogenesis. Thorax.

[63] Onursal C (2021). Collagen biosynthesis, processing, and maturation in lung ageing. Front Med (Lausanne).

[64] Ito S (2017). Biology of Hsp47 (Serpin H1), a collagen-specific molecular chaperone. Semin Cell Dev Biol.

[65] Li Y (2018). Collagen prolyl hydroxylase 3 has a tumor suppressive activity in human lung cancer. Exp Cell Res.

[66] Herrera J (2019). Registration of the extracellular matrix components constituting the fibroblastic focus in idiopathic pulmonary fibrosis. JCI Insight.

[67] Broekelmann TJ (1991). Transforming growth factor beta 1 is present at sites of extracellular matrix gene expression in human pulmonary fibrosis. Proc Natl Acad Sci U S A.

[68] Herrera J (2018). Dicer1 deficiency in the idiopathic pulmonary fibrosis fibroblastic focus promotes fibrosis by suppressing microRNA biogenesis. Am J Respir Crit Care Med.

[69] Booth AJ (2012). Acellular normal and fibrotic human lung matrices as a culture system for in vitro investigation. Am J Respir Crit Care Med.

[70] Yang X (2021). Ppic modulates CCl_4_-induced liver fibrosis and TGF-β-caused mouse hepatic stellate cell activation and regulated by miR-137-3p. Toxicol Lett.

[71] Marchitti SA (2010). Aldehyde dehydrogenase 3B1 (ALDH3B1): immunohistochemical tissue distribution and cellular-specific localization in normal and cancerous human tissues. J Histochem Cytochem.

[72] Klein ME (2018). PDLIM7 and CDH18 regulate the turnover of MDM2 during CDK4/6 inhibitor therapy-induced senescence. Oncogene.

[73] Schafer MJ (2017). Cellular senescence mediates fibrotic pulmonary disease. Nat Commun.

[74] Barnes AM (2006). Deficiency of cartilage-associated protein in recessive lethal osteogenesis imperfecta. N Engl J Med.

[75] Parz NR (2021). Reticulocalbin 3 is involved in postnatal tendon development by regulating collagen fibrillogenesis and cellular maturation. Sci Rep.

[76] Bodempudi V (2014). miR-210 promotes IPF fibroblast proliferation in response to hypoxia. Am J Physiol Lung Cell Mol Physiol.

[77] Shin S (2017). Human steroid sulfatase induces Wnt/β-catenin signaling and epithelial-mesenchymal transition by upregulating Twist1 and HIF-1α in human prostate and cervical cancer cells. Oncotarget.

[78] Annes JP (2004). Integrin alphaVbeta6-mediated activation of latent TGF-beta requires the latent TGF-beta binding protein-1. J Cell Biol.

[79] Fiore VF (2018). αvβ3 Integrin drives fibroblast contraction and strain stiffening of soft provisional matrix during progressive fibrosis. JCI Insight.

[80] Hua R (2021). Small leucine-rich proteoglycans in physiological and biomechanical function of bone. Matrix Biol Plus.

[81] Perkins TN (2021). The perplexing role of RAGE in pulmonary fibrosis: causality or casualty?. Ther Adv Respir Dis.

[82] Kuhn C (1989). An immunohistochemical study of architectural remodeling and connective tissue synthesis in pulmonary fibrosis. Am Rev Respir Dis.

[83] Wilson SE (2021). TGF beta -1, -2 and -3 in the modulation of fibrosis in the cornea and other organs. Exp Eye Res.

[84] Kamp JC (2022). Comparative analysis of gene expression in fibroblastic foci in patients with idiopathic pulmonary fibrosis and pulmonary sarcoidosis. Cells.

[85] Pandit KV (2010). Inhibition and role of let-7d in idiopathic pulmonary fibrosis. Am J Respir Crit Care Med.

[86] Herrera JA MicroRNAs in mechanical homeostasis. Cold Spring Harb Perspect Med.

[87] Perez-Riverol Y (2019). The PRIDE database and related tools and resources in 2019: improving support for quantification data. Nucleic Acids Res.

[88] Tyanova S (2016). The MaxQuant computational platform for mass spectrometry-based shotgun proteomics. Nat Protoc.

[89] UniProt Consortium (2021). UniProt: the universal protein knowledgebase in 2021. Nucleic Acids Res.

[90] https://www.r-project.org/.

[91] Yu G (2016). ReactomePA: an R/Bioconductor package for reactome pathway analysis and visualization. Mol Biosyst.

