# Supplementary material for: The UIP/IPF fibroblastic focus is a collagen biosynthesis factory embedded in a distinct extracellular matrix
Source: JCI Insight. 2022 Aug 22;7(16):e156115. doi: 10.1172/jci.insight.156115 (PMC9462507; doi:10.1172/jci.insight.156115)
Supplement: Supplemental data [file jciinsight-7-156115-s053.pdf]

Supplemental Table 1

A)

Non-fibrotic	1	2	3	4	5	6
Age	84	77	74	52	75	58
Sex	M	M	F	M	M	F
FEV1 %	79	-	54	89	101	111
FVC %	102	-	62	109	112	124

B)

Fibrotic	1	2	3	4	5	6
Age	64	59	60	52	55	61
Sex	F	M	M	F	M	M
FEV1 %	68	57	61	-	64	59
FVC %	63	49	53	76	64	52
DLCO %	30	33	42	25	44	23
Radiology	Atypical UIP	UIP	NSIP	UIP	UIP	NSIP
Pathology	UIP	UIP with granulomas	UIP	UIP	UIP	UIP
Clinical diagnosis	IPF	Sarcoidosis	IPF	IPF	IPF	IPF
Prednisolone	Y	N	N	N	N	N
Pirfenidone	Y	Y	Y	Y	Y	Y
Nintedanib	N	N	N	N	N	N

Supplemental Table 1: Patient Demographics. Non-fibrotic (**A**) and fibrotic (**B**) patient demographics. Lung function for fibrotic patients was taken as the last pulmonary function reading before transplant.

Supplemental Figure 1

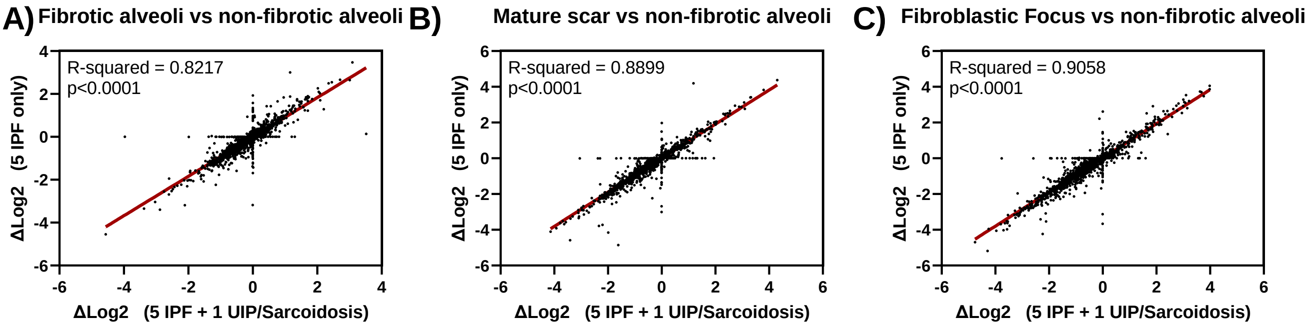

Supplemental Figure 1: Removal of the UIP/sarcoidosis specimen does not dramatically alter protein expression per region. A scatter plot showing the correlation of expression when the UIP/Sarcoidosis specimen was kept (X-axis) or removed (Y-axis) when comparing the (**A**) fibrotic alveoli, (**B**) mature scar, and (**C**) fibroblastic focus versus non-fibrotic alveoli control. The linear regression is presented within a 95% confidence interval.

Supplemental Figure 2

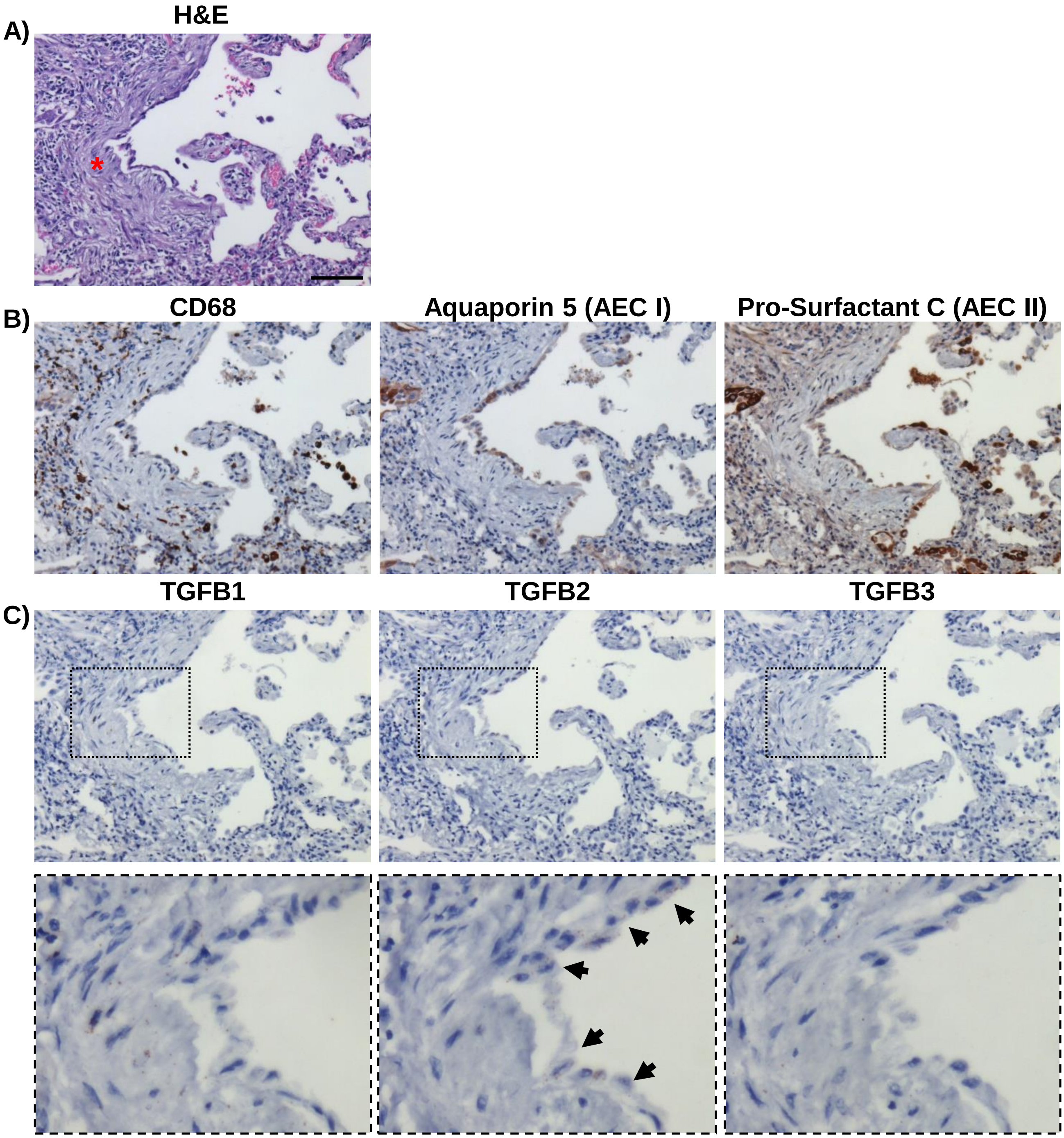

Supplemental Figure 2: Distribution of type I & II alveolar epithelial cells and TGFβ1-3 in the fibroblastic focus and adjacent alveoli. Another UIP/IPF specimen was serially sectioned and histologically stained for (**A**) H&E (red asterisk denotes the fibroblastic focus). (**B**) Immunostained for macrophages (CD68), type I alveolar epithelial cells (anti-aquaporin 5), & type II alveolar epithelial cells (pro-surfactant C, pSC), (**C**) RNA in situ hybridization for TGFβ1-3. Scale bar represents 100 microns (N=5 UIP/IPF specimens, this is a second representative image).

Supplemental Figure 3

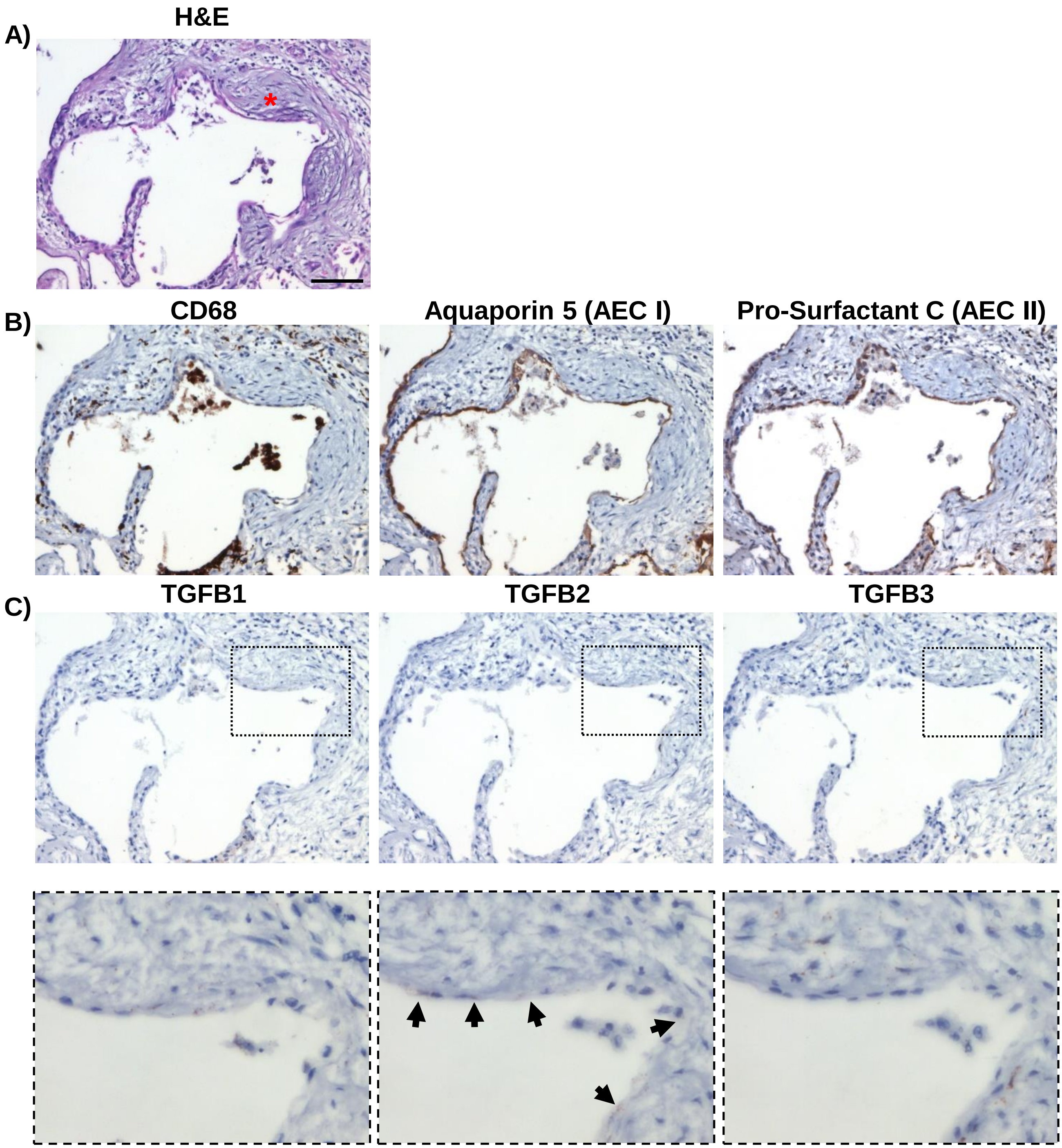

Supplemental Figure 3: Distribution of type I & II alveolar epithelial cells and TGFβ1-3 in the fibroblastic focus and adjacent alveoli. Another UIP/IPF specimen was serially sectioned and histologically stained for (**A**) H&E (red asterisk denotes the fibroblastic focus). (**B**) Immunostained for macrophages (CD68), type I alveolar epithelial cells (anti-aquaporin 5), & type II alveolar epithelial cells (pro-surfactant C, pSC), (**C**) RNA in situ hybridization for TGFβ1-3. Scale bar represents 100 microns (N=5 UIP/IPF specimens, this is a third representative image).

Supplemental Figure 4

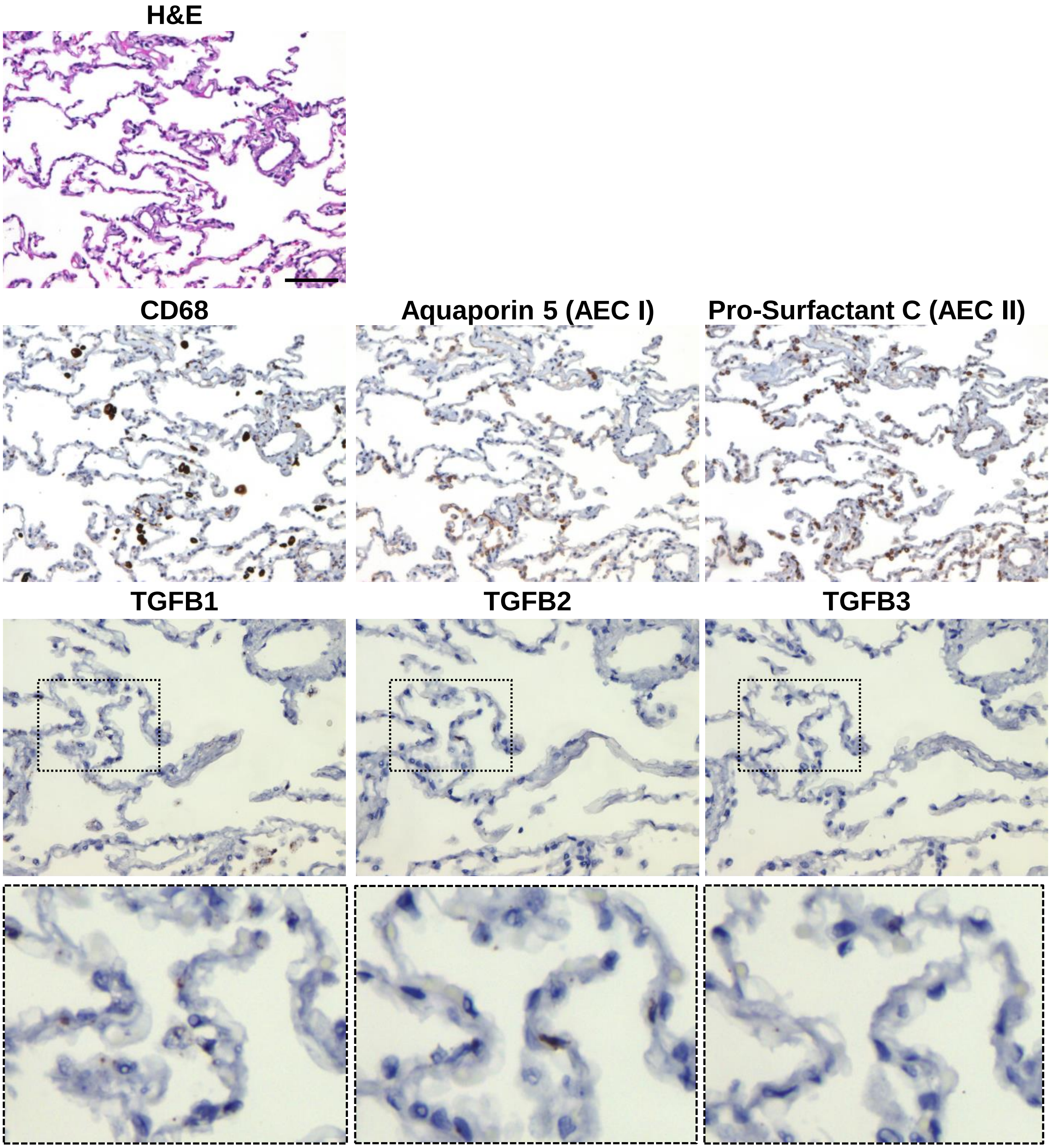

Supplemental Figure 4: Distribution of type I & II alveolar epithelial cells and TGFβ1-3 in non-fibrotic lung. A non-fibrotic specimen was serially sectioned and histologically stained for (**A**) H&E. (**B**) Immunostained for macrophages (CD68), type I alveolar epithelial cells (anti-aquaporin 5), & type II alveolar epithelial cells (pro-surfactant C, pSC) (**C**) RNA in situ hybridization for TGFβ1-3. Scale bar represents 100 microns (N = 4 non-fibrotic specimens, representative image shown)

Supplemental Figure 5

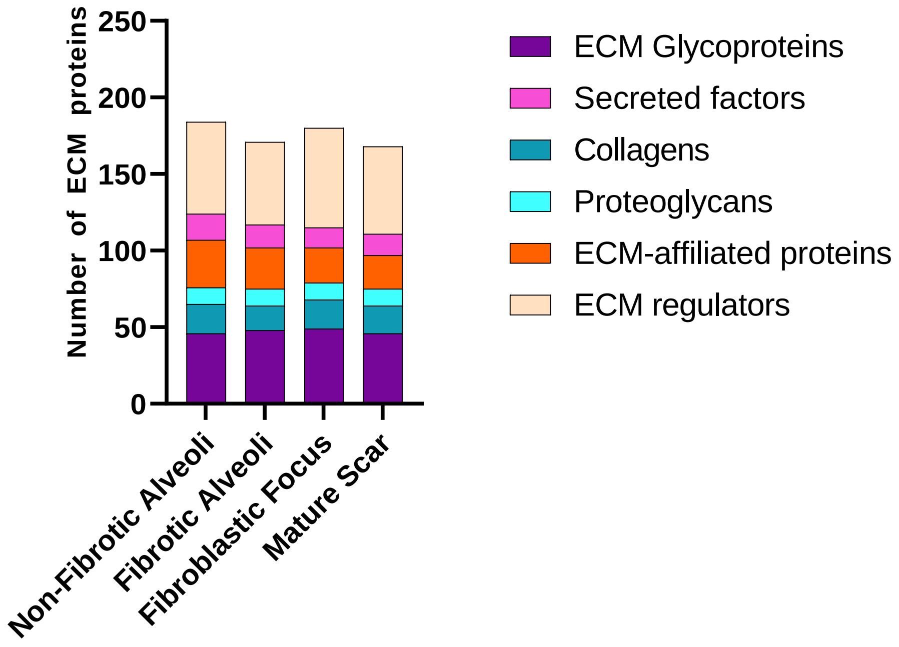

Supplemental Figure 5: Spatial ECM diversity. The detected ECM proteins, in each group, were stratified and graphed into 6 ECM categories: ECM glycoproteins, collagens, proteoglycans, ECM-affiliated proteins, ECM regulators, and secreted factors.

Supplemental Figure 6

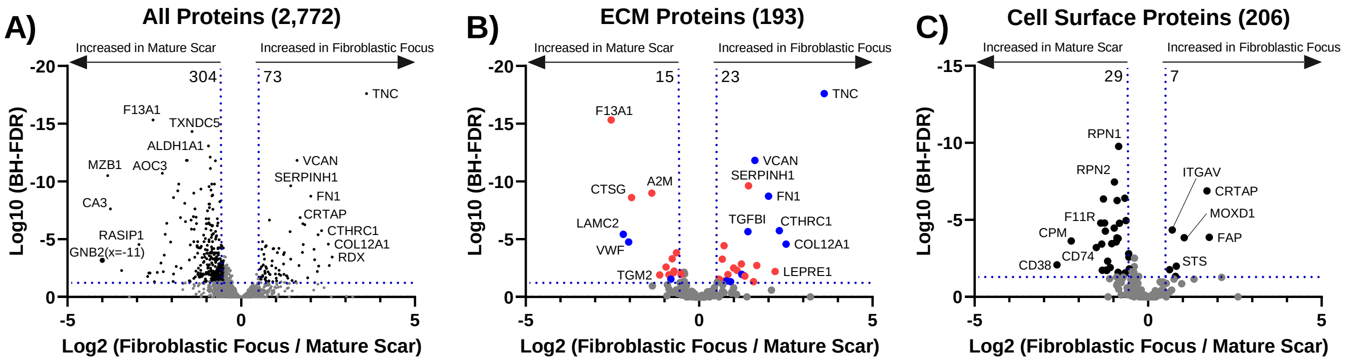

D) Up-regulated in Fibroblastic Focus

Pathway name/reactome pathways identifier (R-HSA)	FDR	Set Size
Extracellular matrix organization/R-HSA-1474244	6.76E-04	12
Collagen biosynthesis and modifying enzymes/R-HSA-1650814	3.89E-03	6
Collagen formation/R-HSA-1474290	1.01E-02	6
Post-translational protein phosphorylation/R-HSA-1474244	1.33E-02	6
Regulation of insulin-like growth factor (IGF) transport and uptake by insulin-like growth factor binding proteins (IGFBPs)/R-HSA-381426	1.76E-02	6

E) Down-regulated in Fibroblastic Focus

Pathway name/reactome pathways identifier (R-HSA)	FDR	Set Size
TCA cycle and respiratory electron transport/R-HSA-1428517	3.91E-04	27
Pyruvate metabolism and Citric Acid (TCA) cycle/R-HSA-71406	3.91E-04	16
Detoxification of reactive oxygen species/R-HAS-3299685	6.55E-04	13
Maturation of spike protein/R-HSA-9694548	6.55E-04	9
Citric acid cycle (TCA)/R-HSA-71403	8.91E-04	11
Glucogeogenesis/R-HSA-70263	2.96E-03	11

Supplemental Figure 6. The fibroblastic focus as compared to mature scar tissue. (**A-C**) Volcano plots comparing the fibroblastic focus to mature scar showing the negative natural log of the false discovery values (FDR) values plotted against the base 2 log (fold change) for each protein. The data in (**A**) is for all proteins, whereas (**B**) was matched against the 'Human Matrisome Project' (http://matrisomeproject.mit.edu) and (**C**) was matched against the 'Cell Surface Protein Atlas' (http://wlab.ethz.ch/cspa/). (**D**) up-regulated or (**E**) down-regulated Reactome pathways for the fibroblastic focus compared to mature scar.

Supplemental Figure 7

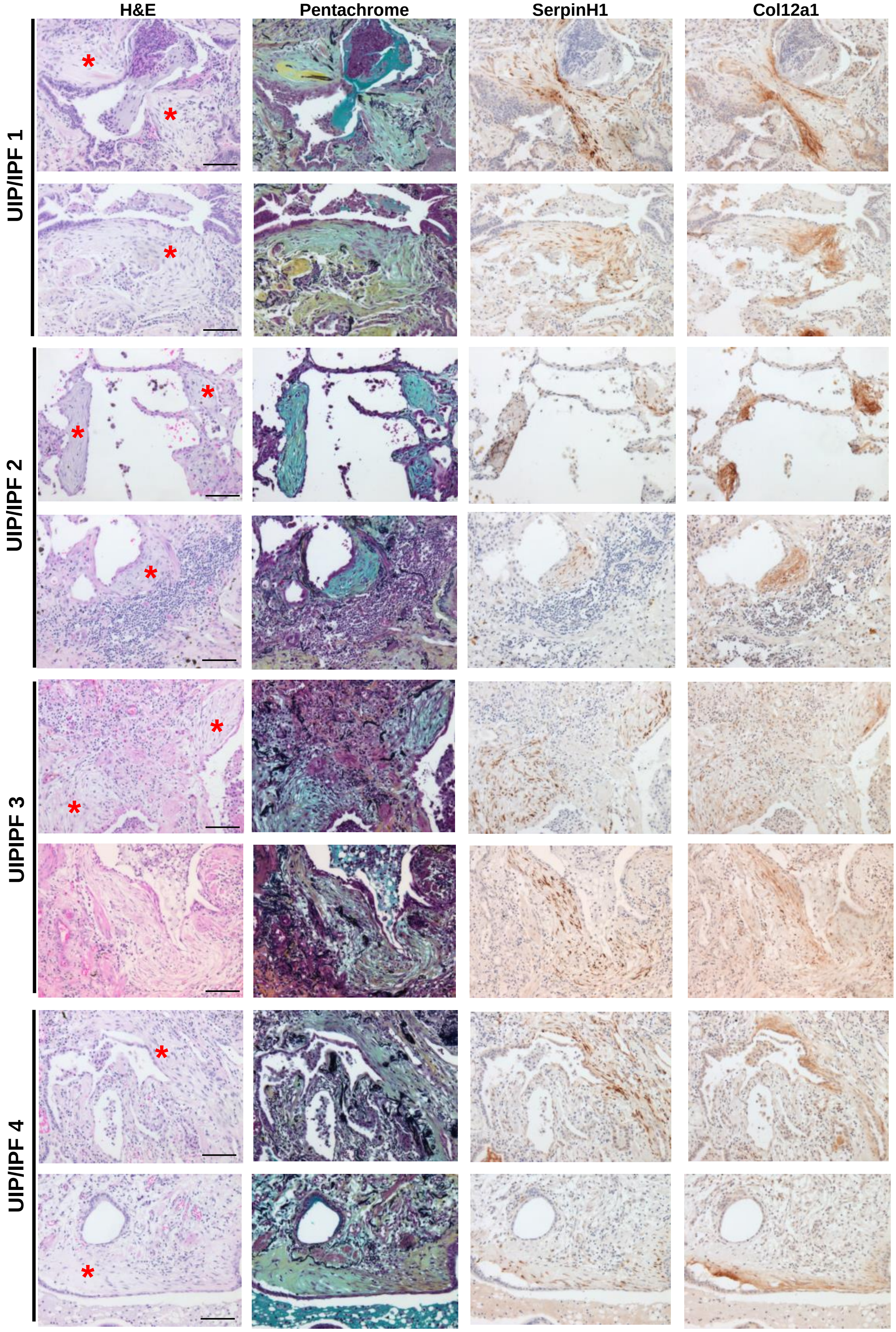

Supplemental Figure 7. SerpinH1 and Col12a1 expression in IPF. 4 UIP/IPF specimens were serially sectioned and stained for H&E, pentachrome, anti-SerpinH1, and anti-Col12a1. We show two additional fibroblastic foci for each specimen (depicted with a red asterisk). Scale bar represents 100 microns.
